# Role of Clathrin and Dynamin in Clathrin Mediated Endocytosis/Synaptic Vesicle Recycling and Implications in Neurological Diseases

**DOI:** 10.3389/fncel.2021.754110

**Published:** 2022-01-18

**Authors:** Kate L. Prichard, Nicholas S. O'Brien, Sari R. Murcia, Jennifer R. Baker, Adam McCluskey

**Affiliations:** Chemistry, School of Environmental and Life Sciences, The University of Newcastle, Callaghan, NSW, Australia

**Keywords:** clathrin mediated endocytosis (CME), clathrin, dynamin, inhibitors, synaptic vesicle recycling (SVR), neurological disorders

## Abstract

Endocytosis is a process essential to the health and well-being of cell. It is required for the internalisation and sorting of “cargo”—the macromolecules, proteins, receptors and lipids of cell signalling. Clathrin mediated endocytosis (CME) is one of the key processes required for cellular well-being and signalling pathway activation. CME is key role to the recycling of synaptic vesicles [synaptic vesicle recycling (SVR)] in the brain, it is pivotal to signalling across synapses enabling intracellular communication in the sensory and nervous systems. In this review we provide an overview of the general process of CME with a particular focus on two key proteins: clathrin and dynamin that have a central role to play in ensuing successful completion of CME. We examine these two proteins as they are the two endocytotic proteins for which small molecule inhibitors, often of known mechanism of action, have been identified. Inhibition of CME offers the potential to develop therapeutic interventions into conditions involving defects in CME. This review will discuss the roles and the current scope of inhibitors of clathrin and dynamin, providing an insight into how further developments could affect neurological disease treatments.

## Introduction

Clathrin mediated endocytosis (CME) is ubiquitous process in all eukaryotic cells. It is the major mechanism for sorting and internalisation of various macromolecules, proteins and lipids and controlling signalling pathway activation. CME plays a key role in the recycling of synaptic vesicles [synaptic vesicle recycling (SVR)] in the brain, allowing the continued signalling across synapses that is vital for intracellular communication in the sensory and nervous systems. Our focus is on the small molecules published to date as potential probe molecules. Major aspects of the biology roles of clathrin mediated endocytosis, we expect are covered within other manuscripts within this issue.

In CME, clathrin polymerises to act as a coat to mediate the internalisation of hormones, nutrients and receptors, while dynamin mediated scission of the vesicle by self-assembling into rings forming a collar around the neck of the vesicle (Royle, 2006; Anggono and Robinson, 2009; Robinson, 2015; Kaksonen and Roux, 2018; Milosevic, 2018). Dynamin and clathrin are vital to CME and play a role in the efficient recycling of synaptic vesicles. SVR mediates the proper function of nervous and sensory systems. These proteins are believed to be contribute towards multiple neurological conditions including, but not limited to, epilepsy (Chin et al., 1995; Di Paolo et al., 2002; Kim et al., 2002), schizophrenia (Schubert et al., 2012), Huntington's (McAdam et al., 2020), Parkinson's (Inoshita and Imai, 2015; Vidyadhara et al., 2019), and Alzheimer's disease (Rafii and Aisen, 2009; Wu and Yao, 2009; Palmer, 2011; Alsaqati et al., 2018). It is highly unlikely, given the complex nature of these conditions, that CME inhibition or defect is the sole disease progenitor. This review will provide an overview of selected health conditions linked with defects in CME/SVR and discuss the current scope of inhibitors of clathrin and dynamin. It is not intended to be a comprehensive listing of human diseases that maybe associated with endocytosis, but to provide an insight into how further development of these compounds could potentially affect the treatment of neurological diseases.

Given that of all the proteins involved in endocytosis, clathrin and dynamin are essentially the only two systems to which there have been reported small molecule inhibitors, our focus is centred on these two proteins. This work describes the structure and function of dynamin and clathrin in endocytosis across clathrin mediated and synaptic vesicle endocytosis; the potential role of SVE in neurological diseases and the current state of the art in developing CME (and SVE) inhibitors via targeting of dynamin and clathrin.

## Clathrin—Structure and Function

**Figure 1:**
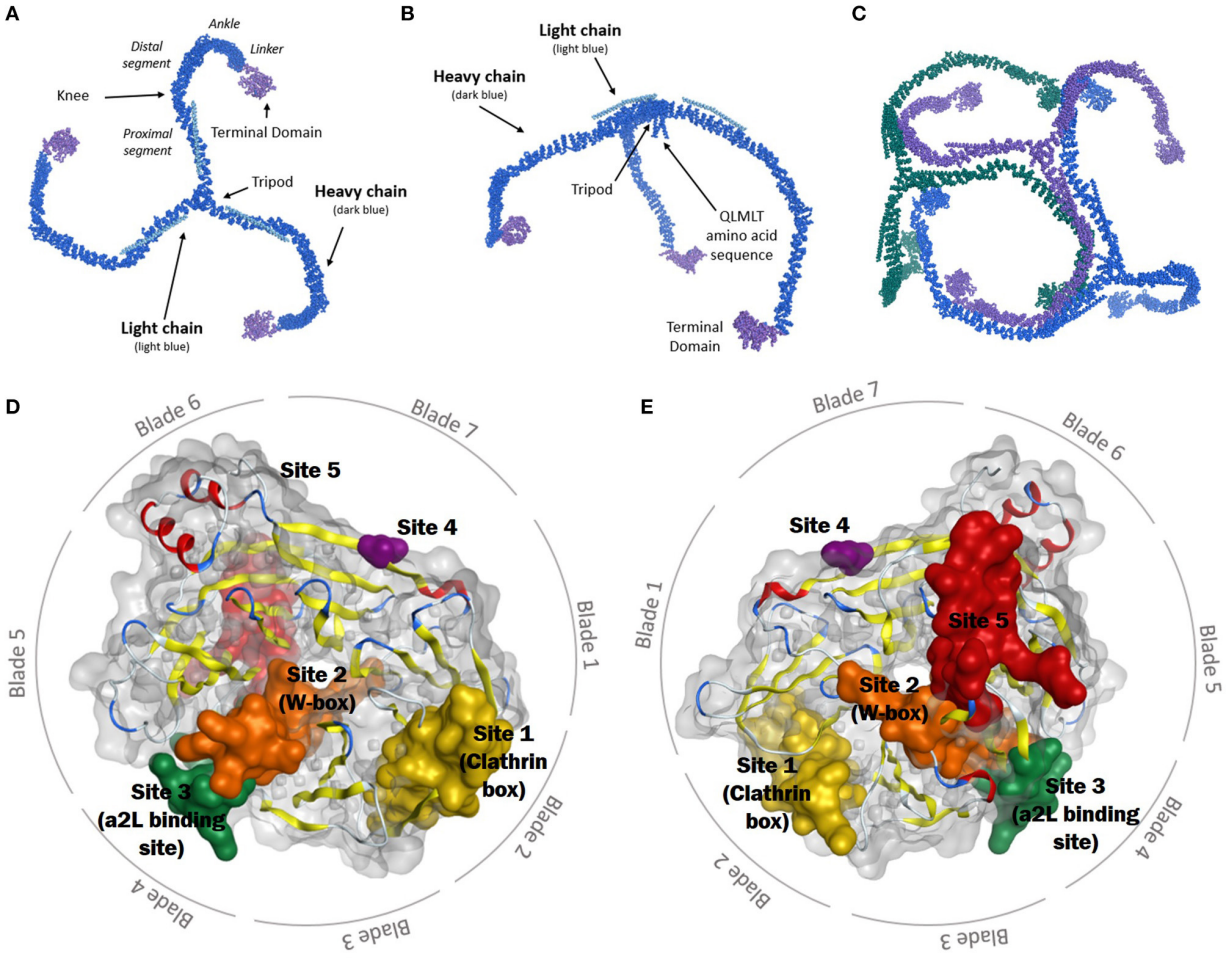
**(A)** Schematic representation of the clathrin triskelion (top view). The heavy chain is displayed in dark blue, the light chain in light blue, and the terminal domain in purple. **(B)** Side view of clathrin triskelion showing the location of the tripod and QLMLT amino acid sequence at the central vertex. **(C)** Schematic representation of three clathrin triskelions bound in lattice formation. Each singular triskelion represented in a different colour (blue, green, and purple) (Fotin et al., 2004a). **(D)** Top view of the CTD, consisting of seven blades. The binding sites are shown: Yellow: Site 1 (clathrin box), orange: Site 2 (W-box), green: Site 3 (aL2 binding site), purple: Site 4, red: Site 5. **(E)** Bottom view of the CTD, showing the seven blades and five binding sites (Alsaqati et al., 2018).

Clathrin, unsurprisingly, is critical to achieving a successful CME outcome (Roth and Porter, 1964; Pearse, 1975). It is principally involved in CME, mitosis, and SVR (Kaksonen and Roux, 2018; Mettlen et al., 2018). Clathrin activity requires the assembly of a macromolecular complex, a triskelion. Each leg is of ca 475Å length and 20Å thickness and radiates from a central hub which contains a helical tripod and QLMLT amino acid sequence (Figures 1A,B) (Ungewickell et al., 1981; Kirchhausen et al., 2014). The clathrin heavy chain (CHC) makes up the length of each leg and associates with a clathrin light chain (CLC) (Kaksonen and Roux, 2018). In humans, two CHC isoforms (CHC17 and CHC22) exist, with 85% amino acid homology. CHC17 is ubiquitous and involved in membrane trafficking and mitosis, while CHC22 is located within skeletal muscle (Kirchhausen et al., 2014; Kaksonen and Roux, 2018). Two 60% homologous CLCs exist, both can associate with CHC17 but not with CHC22 (Royle, 2006; Kirchhausen et al., 2014; Kaksonen and Roux, 2018). The CLC binds to the CHC at a series of zig-zags, where hydrophobic residues of the CLC face the surface of the heavy chain. The CLC has two flexible regions, which lie near the curved knee and the central vertex of the CHC when bound. The C-terminal of the CLC sits close to the vertex of the CHC triskelion (Kirchhausen and Toyoda, 1993; Kirchhausen et al., 2014).

The CHC comprises six segments; proximal, knee, distal, ankle, linker and terminal domain (Figure 1A) (Kirchhausen et al., 2014). The whole leg adopts a stacked hairpin structure containing a ~145-residue motif of five hairpins, repeated seven times (Robinson, 2015). These legs are flexible, to allow the formation of differing diameter coats of hexagons and pentagons by the polymerisation of clathrin triskelions (Kirchhausen, 2000). In the lattice, the proximal segment of each leg passes beside the proximal segment of the adjacent triskelion, the central vertex forming the points of the lattice, with the contour of each leg spiralling inwards and the terminal domain sitting towards the inside edge of the lattice (Figure 1C) (Fotin et al., 2004a; Kirchhausen et al., 2014; Robinson, 2015; Kaksonen and Roux, 2018).

### Clathrin Terminal Domain

The clathrin terminal domain (CTD) (resides 1-330) comprises seven β-sheets, stacked around a central axis in a propeller-like structure (Figures 1D,E) (Ter Haar et al., 1998). The propeller has an elliptical cross section due to increased spacing between blades 1 and 7, and 3 and 4, occupied with short helical segments. Differences in inter-strand loops gives rise to variation in the number of residues in each blade (Robinson, 2015). The terminal domain projects inwards, towards the membrane allowing interactions with adaptor proteins at a number of binding sites to direct incorporation of cargo (Table 1 details the key binding residues at each binding site). Adaptors bind to the CTD via short linear peptide sequences (Collette et al., 2009; Robinson, 2015).

**Table 1:** Summary of the currently known and proposed binding sites of the clathrin terminal domain, detailing key residues, and binding examples.

**Site**	**Adaptor motif**	**Key CHC residues**	**Binding examples**	**References**
1	LΦXΦ [DE]	I80, T87, Q89, F91, K96, K98	Amphiphysin	Collette et al., 2009; Lemmon and Traub, 2012; Muenzner et al., 2017
			β-arrestin 1	
			AP-2 β2 subunit	
2 (W-Box)	PWXXW	F27, Q152, I154, I170	Amphiphysin	Ramjaun and Mcpherson, 1996; Miele et al., 2004; Collette et al., 2009; Chen et al., 2020
			SNX9	
3	[LI][LI]GXL	R188, Q192	β-arrestin iL	Kang et al., 2009; Muenzner et al., 2017
			AP-2 β2 subunit	
4	Unknown	E11	Unknown	Willox and Royle, 2012
5	Unknown	N175, G179, R221, Q23, F252, F260	Unknown	Abdel-Hamid and McCluskey, 2014; Ghods et al., 2018
Ankle	Unknown, non-linear	C682, G710	GGA1	Knuehl et al., 2006

The CTD is connected to the distal leg by a short zig-zag linker of alpha helices followed by the ankle region (Lemmon and Traub, 2012; Kirchhausen et al., 2014). The ankle also contains the binding site of ADP-ribosylation factor-binding protein (GGA1) and adaptor protein (AP) 1 and AP-2 beta chains (Knuehl et al., 2006). This binding site has an unknown non-linear motif, but contains key residues C682, G710 (Knuehl et al., 2006).

### Site 1 (Clathrin Box)

Site directed mutagenesis of clathrin determined an interaction with β-arrestin and arrestion3, where the first 100 residues were sufficient for binding *in vitro*. This fragment corresponds to blades 1 and 2 of the propeller, with key residues (Q89, F91, K96, K98) found to lie in the groove between the blades (Figures 1D,E) (Ter Haar et al., 1998; Collette et al., 2009). This is Site 1, also known as the clathrin box. The principle contact sites gave a sequence known as the clathrin box motif; LΦXΦ[DE]where Φ is a hydrophobic reside and alternative residues are bracketed, [] (Lemmon and Traub, 2012). The β-2 subunit of adaptor protein 2 (AP-2) also contains this motif, which overlaps with another clathrin binding motif for site 3 possibly allowing AP-2 binding at both sites 1 and 3 (Muenzner et al., 2017).

### Site 2 (W-Box)

A second binding site on clathrin, the W-box (Ramjaun and Mcpherson, 1996) site, binds proteins containing the W-box motif; PWXXW (where X is any amino acid), such as amphiphysin and sorting nexin 9 (SNX9) (Royle, 2006; Popova et al., 2013). Located in the deep pocket at the centre of the top surface the key resides of the W-box include F27, Q152, I154, and I170 (Figures 1D,E) (Miele et al., 2004; Collette et al., 2009; Chen et al., 2020).

### Site 3 (a2L Binding Site)

Site 3, the aL2 (arrestin 2L) binding site is a shallow hydrophobic groove between blades 4 and 5 defined by W164, L183, R188, V190, Q192, I194, 231, E232, T235, and K245 (Figures 1D,E). The binding motif [LI][LI]GXL, observed in arrestin 2L has also been found in other trafficking accessory proteins such as the adaptins subunits of AP-1 and AP-2 (Muenzner et al., 2017), synaptotagmin and synaptojanin, however it is currently unknown if these proteins use this motif to interact with clathrin (Kang et al., 2009).

### Site 4

A triple knockout of sites 1–3, comprising a 2 point mutations in the W-box (Q152L, Il54Q), and a2L binding site (R188A, Q192A) and a 4 point mutation in the clathrin box (T87A, Q89A, K96E, K98E) retained CME function, suggesting the presence of a fourth binding site on the CTD (Willox and Royle, 2012). A region on the surface exposed blade 7 at E11, was identified as site 4, although the binding motif and protein partners are currently unknown (Figures 1D,E) (Willox and Royle, 2012).

### Site 5

**Figure 2:**
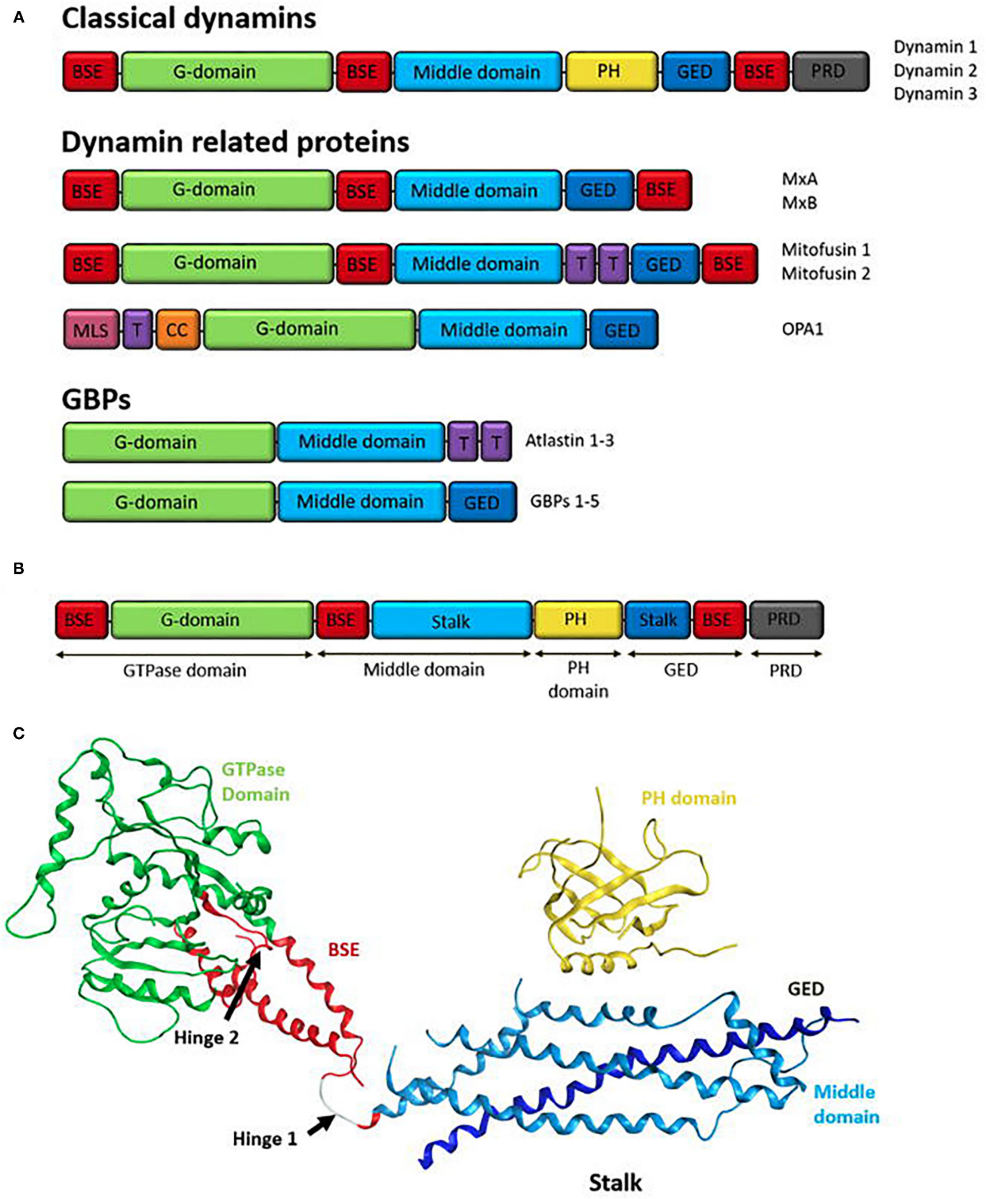
**(A)** Domain structure of the human dynamin superfamily. All DSPs contain a GTPase domain, a middle domain (MD), and a GTPase effector domain (GED). The MD and GED comprises the stalk region of the dynamin structure. Additionally, most dynamins contain a domain for interactions with lipid membranes, such as a pleckstrin-homology (PH) domain or trans membrane domain (T). Classical dynamins contain a PH domain and also a C-terminal proline-rich domain (PRD). Other family members may have different sequences at the N-terminal or in between the MD and GED domains. Human dynamin superfamily members are grouped according to their domain structure into; classical dynamins (dynamin 1-3), Dynamin related proteins (examples shown; MxA/MxB, Mitofusin 1 and 2, and OPA1), and guanylate-binding proteins GBPs (examples shown; Atlastin 1-3 and GBP 1-5). Structural components are colour coordinated; red: bundle signalling element (BSE), green: G-domain, light blue: middle domain, yellow: PH domain, dark blue: GED, purple: transmembrane domain (T), grey: PRD (Praefcke and McMahon, 2004; Ford et al., 2011), pink: mitochondria localisation signal (MLS) and orange: coiled coil motif (CC). **(B)** Domain Structure of human dynamin 1 (Ford et al., 2011). Classical domain names are indicated below. **(C)** Ribbon representation of human dynamin 1. Domains represented by colours coordinating to the domain structure in A. GTPase domain (green), BSE (red), stalk region consisting of the middle domain (light blue) and GED (dark blue) and the PH domain (yellow). Hinge 1 and 2 are labelled (Ford et al., 2011).

A fifth proposed site lies between blades 5 and 6, lined with residues N175, G179, R221, Q23, F252, and F260 (Figure 2B). This site was suggested by a molecular dynamics investigation of the potential binding poses of the marine sesquiterpenoid, bolinaquinone. In a protein-pull down experiment, bolinaquinone was reported as a specific inhibitor of clathrin (Abdel-Hamid and McCluskey, 2014; Ghods et al., 2018).

## Dynamin—Structure and Function

Dynamin, a 100-kDa large GTPase and mechanochemical enzyme, is recruited post clathrin coated vesicle formation and is critical to vesicle scission and ultimately recycling of the clathrin coated pits (Robinson et al., 1987; Shpetner and Vallee, 1989; Popova et al., 2013). Dynamin differs significantly from traditional GTPases (Obar et al., 1990), and is the founding member of the dynamin superfamily of large GTPases (Anggono and Robinson, 2009). Dynamin, in addition to its CME role (Praefcke and McMahon, 2004), in involved in SVR (Ferguson and De Camilli, 2012; Antonny et al., 2016), caveolae internalization (Oh et al., 1998), and possibly vesicle trafficking in and out of the Golgi (Llorente et al., 1998; Nicoziani et al., 2000).

### Dynamin Like Proteins

Dynamin spans species, from bacteria to human (Hinshaw, 2000; Anggono and Robinson, 2009; Ramachandran and Schmid, 2018). Dynamin and dynamin-related proteins (DRPs) are distinguished from traditional GTPases by a larger GTPase domain, their unique ability to self-assemble and disassemble–to which their GTP hydrolysis mechanism is distinctly associated (Hinshaw and Schmid, 1995; Marks et al., 2001; Chappie et al., 2010; Faelber et al., 2011). They have low GTP-binding affinities (Hinshaw and Schmid, 1995; Gasper et al., 2009), high GTPase activity and the ability to bind and tubulate lipid membranes (Takei et al., 1995; Hinshaw, 2000; Danino et al., 2004; Roux et al., 2006).

Structurally the dynamin superfamily proteins (DSPs) are classified as: the *classical dynamins* (Nakata et al., 1993; Sontad et al., 1994; Gray et al., 2003), the *dynamin-related proteins* (DRPs) and the *guanylate-binding proteins* (GBPs)/atlastins (Prakash et al., 2000a,b). The DRPs include dynamin-like proteins (Dlp) (Labrousse et al., 1999), Mx proteins (MxA, MxB) (Staeheli et al., 1986; Janzen et al., 2000; Kochs et al., 2002), optic atrophy 1 (OPA1) (Olichon et al., 2002; Satoh et al., 2003) and mitofusins (Rapaport et al., 1998; Santel and Fulller, 2000) (Figure 2). Many DSPs are involved in membrane re-modelling events including fission, fusion and intercellular tracking of vesicles and large organelles. Others such as the Mx proteins have roles independent of membrane properties like viral resistance to host cells (Ramachandran and Schmid, 2018). DLPs, also known as non-classical dynamins, are believed to assist in the recruitment of the classical dynamins to the membrane for vesicle scission (Singh et al., 2017).

Common to all DSPs are the large N-terminal GTPase domain (~300 amino acids), the middle domain (MD), and the GTPase effector domain (GED) (Figure 2A). Most DSPs contain additional domains that are tailored to their specific cellular functions or subcellular location. In the classical dynamins, the three basic set of domains is supplemented by two additional functional domains—a pleckstrin-homology (PH) domain and a proline-rich domain (PRD) at the C-terminus (Praefcke and McMahon, 2004; Anggono and Robinson, 2009).

### Classical Dynamins

There are three mammalian dynamin genes—dynamin 1, 2, and 3 which are known as the “classical dynamins” (Cao et al., 1998; Praefcke and McMahon, 2004). These dynamins share the same domain organisation and 80% overall homology, but they have distinctive expression patterns, with variation between isoforms primarily observed within the PRD (Ferguson and De Camilli, 2012; Jimah and Hinshaw, 2019).

Dynamin 1 is predominately expressed at high levels in the central nervous system, where it is concentrated in the presynaptic nerve terminals. Although not generally not present in non-neuronal tissues (Nakata et al., 1991; Ferguson et al., 2007; Anggono and Robinson, 2009), dynamin 1 has been detected in cultured cell lines (Liu et al., 2008; Ferguson et al., 2009) and in the testes (Zhou et al., 2017). In the brain, dynamin 1 is expressed at ~100-fold greater than other isoforms and in fact, is among the most abundant neuronal proteins in the brain (Anggono and Robinson, 2009). Dynamin 2 is ubiquitously expressed in all tissues where it plays a major role in mitosis (Nakata et al., 1991; Cook et al., 1994; Chircop et al., 2011; Smith and Chircop, 2012). Dynamin 3 is found predominantly in the testes and brain, where it is found both pre- and post- synaptically, and in low levels in the lungs (Nakata et al., 1993; Cao et al., 1998; Gray et al., 2003; Raimondi et al., 2011; Ferguson and De Camilli, 2012).

All three dynamins play a role in vesicle scission during the specialised neuronal function of rapid SVR (Anggono and Robinson, 2009). Dynamin 1 mediates the majority of synaptic vesicle endocytosis (SVE), particularly during a depolarization stimulus (Anggono and Robinson, 2009; Clayton et al., 2009) where dynamin 1 and several other endocytic proteins are constitutively phosphorylated in resting synapses and upon nerve stimulation. In addition to the role in CME, dynamin also plays a role in clathrin independent synaptic vesicle recycling pathways including clathrin independent fast endocytosis, fast endophilin mediated endocytosis and ultrafast endocytosis, where dynamin is involved in both scission and membrane curvature (Chanaday et al., 2019; Renard and Boucrot, 2021). Dynamin 1 is rapidly dephosphorylated (at S774 and S778) by the calcium and calmodulin-dependent phosphatase calcineurin (Cousin and Robinson, 2001) which is predicted to facilitate dynamin interactions with endocytic proteins to promote endocytosis (Anggono et al., 2006), specifically this dephosphorylation is believed to play a role in triggering activity dependent bulk endocytosis (Anggono et al., 2006; Cheung and Cousin, 2013). Phosphorylation and dephosphorylation may also play a role in regulation of activity in dynamin 1. Up to a 12-fold increase in GTPase activity has been shown to be caused by phosphorylation of dynamin 1 by protein kinase C, while the dephosphorylation results in inhibition of GTPase activity. Dephosphorylation also directs dynamin to the membrane, and the inhibition of GTPase activity may allow time for binding at the neck of a forming vesicle prior to GTPase hydrolysis to facilitate scission (Hinshaw, 2000).

Dynamin 2 plays a major role in SVR, mainly in the slow endocytosis that occurs after nerve stimulation has ceased (Anggono and Robinson, 2009). In neurons and non-neuronal cells, dynamin 2 mediates CME and mitosis (Chircop et al., 2011; Smith and Chircop, 2012; Kaksonen and Roux, 2018; Mettlen et al., 2018; Bhave et al., 2020). Dynamin 2 also has roles in caveolae budding (Henley et al., 1998; Oh et al., 1998), phagocytosis (Gold et al., 1999), podosome formation (Ochoa et al., 2000) and in endocytosis-independent cellular functions, such as actin dynamics (Orth and McNiven, 2003) and cytokinesis (Van Dam and Stoorvogel, 2002) and mitosis (Chircop et al., 2011; Smith and Chircop, 2012).

Dynamin 3 is proposed to have a specific postsynaptic role, forming specialised endocytic sites that locally recycle AMPA (α-amino-3-hydroxy-5-methyl-4-isoxazole propionic acid) receptors found in dendritic spine tips (Lu et al., 2007). This is consistent with a report of dynamin 3 binding to the ENA-VASP-homology 1 (EVH1) domain of the postsynaptic protein Homer through a PXXF motif (Gray et al., 2003). This motif is not present in dynamin 1 and dynamin 2. This preferential enrichment was not confirmed by another study which highlighted the overlapping presynaptic roles of dynamin 3 with dynamin 1 (Raimondi et al., 2011). Both dynamin 1 and 3 play critical roles in neurotransmission with individual conditional dynamin knockouts in mice having no effect on the development nerve terminals in mouse brain (calix of Held). A combined dynamin 1 and 3 knockdown results in a progressive synaptic transmission loss. This suggests that one or more of dynamin 1, 2 and 3 can rescue synaptic transmission caused by deletion of one dynamin isoform, but not of two. In turn this suggests a degree of functional dynamin isoform redundancy.

### Structure

Classical dynamins comprise an extended structure with five domains; amino-terminal GTP hydrolysis (GTPase) domain, a “middle” domain (MD), a pleckstrin-homology (PH) domain, a GTPase effector domain (GED), and a proline-rich carboxy-terminal (PR) domain (Figure 2B) (Hinshaw, 2000; Ford et al., 2011; Ferguson and De Camilli, 2012; Kong et al., 2018; Jimah and Hinshaw, 2019).

The GTPase domain sits on a long helical “stalk” composed of anti-parallel helices from the middle domain to the N-terminal region of GED (Chappie et al., 2010, 2011; Faelber et al., 2011), linking the GTPase domain to the lipid interacting domains and providing interfaces for higher-order assembly (Jimah and Hinshaw, 2019). The PH domain forms the vertex or “foot” of the stalk hairpin and binds membranes (Ferguson et al., 1994). Linking the GTPase domain and “stalk” is a α-helical bundle, referred to as the bundle signalling element (BSE) (Chappie et al., 2010) or neck (Low and Löwe, 2006), which is formed from non-contiguous sequences at the N- and C-terminal helix of the GTPase domain, as well as the carboxy terminal of the GED (Chappie et al., 2010; Ramachandran and Schmid, 2018; Jimah and Hinshaw, 2019). The PRD domain emerges at the boundary between BSE and the GTPase domain and is expected to be unfolded, most likely projecting away from the membrane where it might interact with other proteins (Figure 2C) (Ferguson and De Camilli, 2012).

### GTPase Domain

The GTPase domain is the most highly conserved region of DSPs (Warnock and Schmid, 1996; van der Bliek, 1999; Anggono and Robinson, 2009), but at ~300 residues is considerably larger than traditional GTPases. The GTPase domain includes splice variations that confer DSP-specific biochemical and biophysical properties and as well as biological functions (Vetter and Wittinghofer, 2001; Ramachandran and Schmid, 2018). The DSP GTPase domain is activated by a nucleotide-dependent dimerization (GAD), thus does not require guanine-exchange factors (GEFs) or GTPase-activating proteins (GAPs), as required by Ras GTPases (Gasper et al., 2009; Chappie et al., 2010). This is due to their unusually high GTPase activity and low affinity for GTP (Praefcke and McMahon, 2004).

Four GTP-binding motifs (G1-G4) exist in the GTPase domain: G1, or P-loop, binds the phosphate; a G2 threonine enables GTP hydrolysis by coordinating a Mg^2+^; G3 also coordinates the Mg^2+^ ion via aspartate and glycine binds the γ-phosphate; and G4 binds the nucleotide base (Niemann et al., 2001; Bramkamp, 2012). The conservation of these motifs is absolute except for the G4 motif in guanylate-binding proteins (Praefcke and McMahon, 2004). K44A, T65A, T141D, and K142A GTPase domain mutations inhibit endocytosis (Damke et al., 1994; Marks et al., 2001; Song et al., 2004).

### Bundle Signalling Element

Several DSPs have two elongated α-helical bundle domains (the BSE), adjacent to the GTPase domain (Jimah and Hinshaw, 2019). The BSE is composed of helices derived from the N- and C-terminal of the GTPase domain and the C-terminal of GED (Ramachandran and Schmid, 2018). In fission, the BSE connects the GTPase domain to the membrane-distal end of the stalk and undergoes a swivel-like motion relative to the GTPase domain during GTP hydrolysis cycles. In fusion, DSPs contain a functionally equivalent nucleotide-responsive flexible hinge in the bipartite helical-bundle (HB) stalk that bounds directly against the GTPase domain (Ramachandran and Schmid, 2018). These conformational shifts are believed responsible for the DSP power stroke in fission and fusion (Chappie et al., 2009, 2010; Yan et al., 2018). An A738T mutation in the BSE suppresses the temperature-sensitive *Drosophila Shibire* mutants which occur within the GTPase domain and cause defects in GTP binding and endocytosis (Kosaka and Ikeda, 1983; Ramaswami et al., 1993; Narayanan et al., 2005).

### Middle Domain

The middle domain (MD) of dynamin lacks sequence homology to any known protein structural motif (Hinshaw, 2000; Anggono and Robinson, 2009). This domain is involved in the formation of dynamin tetramers required for the higher-order self-assembly of tetramers into rings and helices (Faelber et al., 2011; Reubold et al., 2015; Kong et al., 2018). The middle domain comprises a N-terminal and C-terminal region (Muhlberg et al., 1997). Between dynamin isoforms the N-terminal region significantly more conserved than the C-terminal region (Warnock and Schmid, 1996). The C-terminal region accounts for the alternative splice sites for all three classical dynamins. A synthetic equivalent of the N-terminal region adopts an α-helical coiled-coil structure that forms stable tetramers in solution and has been implicated in dynamin-dynamin assembly (Okamoto et al., 1999; Smirnova et al., 1999). It also makes extensive contacts with the self-assembling GED (Anggono and Robinson, 2009).

### GTPase Effector Domain

The GED, or coiled-coil domain is involved in protein-protein interactions (Lupas et al., 1991; Okamoto et al., 1999). This domain binds to the GTPase domain, to the MD, and to itself to form homodimers (Muhlberg et al., 1997; Okamoto et al., 1999; Zhang and Hinshaw, 2001). These interactions drive dynamin self-assembly. The addition of an isolated GED to an unassembled dynamin stimulated its GTPase catalytic activity by up to 100-fold. The GED acts as a dynamin GAP, stimulating both dynamin assembly and GTPase activity (Muhlberg et al., 1997; Sever et al., 1999). The GED, along with the middle domain, make up the “stalk.”

### Pleckstrin-Homology Domain

Among the DSPs only the classical dynamins contain the PH domain (Anggono and Robinson, 2009). This domain contains a seven stranded β-sheet sandwich, with a dynamin hairpin, and three variable loops, ending in the C terminal α-helix (Ferguson et al., 1994; Timm et al., 1994). The PH domain exhibits high homology with pleckstrin, a major PCK substrate in platelets and is found in many proteins (Hinshaw, 2000). Most variation is observed in the three (variable) loops. Each presents a predominantly hydrophobic region, to promote membrane interactions, polymerisation dynamins and membrane curvature with a positive charge within the binding site (Ramachandran et al., 2009; Liu et al., 2011). Dynamins PH domain shows low lipid specificity, but favours binding to phosphatidylinositol-4,5-biphosphate (PI(4,5)P_2_) (Ferguson et al., 1994; Zheng et al., 1996; Anggono and Robinson, 2009). PI(4,5)P_2_ binding to dynamin produces the highest activation of its GTPase activity. Lipid binding is significantly enhanced when the PH domain is oligomerized (Salim et al., 1996; Klein et al., 1998). Dynamin with PH-domain mutants that impair phosphoinositide binding (PI(4,5)P_2_) exert dominant negative effects on CME (Lee et al., 1999; Vallis et al., 1999). The PH domain is required for membrane localisation and CME.

### Proline-Rich Domain

Only classical dynamins possess a PRD. Comprising several BAR and SH3 domain binding sites (defined by a PXXP motif), it is a protein-protein interaction domain for a variety of signalling and cytoskeletal proteins (Mcpherson, 1999; Simpson et al., 1999; Anggono and Robinson, 2009; Ramachandran and Schmid, 2018). These function to recruit dynamin to endocytic sites and coordinate dynamin's function with these other factors during endocytosis (Shpetner et al., 1996; Lundmark and Carlsson, 2004; Anggono et al., 2006; Anggono and Robinson, 2009). For example, amphiphysin directs dynamin to the coated pits by binding to both dynamin and the AP-2 α-subunit complex (David et al., 1996; Shupliakov et al., 1997). Dynamin lacking the PRD cannot rescue endocytic defects in dynamin-knockout fibroblasts (Ferguson et al., 2009). A major function of PRD is to target dynamin to its site of action in the cell. Overexpression of Dyn1ΔPRD results in a failure of dynamin to accumulate at clathrin coated pits (CCP) (Anggono and Robinson, 2009). The PRD also enables dynamin recruitment by key partners such as syndapin that may be involved in membrane tubulation and at the synapse (Qualmann et al., 1999). Dynamin 1 PRD is also the site for its phosphorylation and regulation of its activity in nerve terminals (Anggono et al., 2006).

### Assembly

**Figure 3:**
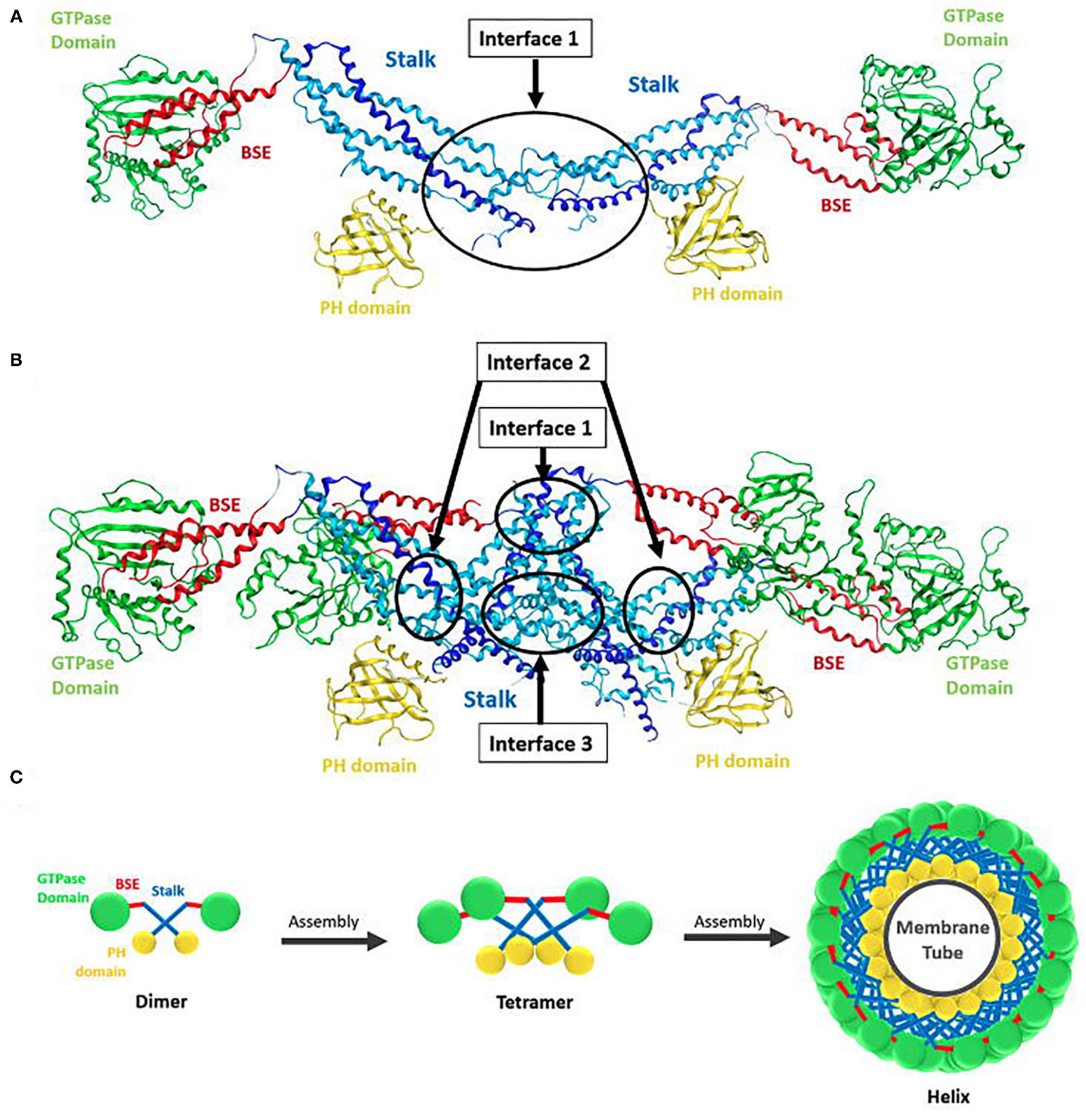
Structure of dynamin dimer and tetramer. **(A)** Crystal structure of a dimer showing the stalk interface (interface 1) needed for self-assembly (Reubold et al., 2015). **(B)** Crystal structure of a tetramer showing the stalk interfaces (interfaces 1, 2, and 3) needed for self-assembly. GTPase domain (green), BSE (red), stalk region consists of the middle domain (light blue) and GED (dark blue) and the PH domain (yellow) (Reubold et al., 2015). **(C)** Structure of dynamin and its assembly into oligomers. Ball and stick representation of a dynamin dimer and tetramer, showing how the dimer polymerize into a tetramer and how tetramers further assemble into a helix around a membrane tube. The GTPase domain is represented in green, BSE in red, stalk in blue, and PH domain in yellow (Chen et al., 2020).

Dynamin self-assembles *in vitro* into rings and helices (Hinshaw and Schmid, 1995). Cryogenic electron microscopy revealed that the dynamin polymer unit is an anti-parallel dimer, with two GTPase domains linked to one side of the cross and the PH domains linked to the other side (Antonny et al., 2016; Kong et al., 2018). Dimerization is mediated by the stalks (MD and GED), which form the cross and provides the interface for further polymerisation (Figure 3). While the simplest dynamin unit is the dimer, in solution dynamin was found to exists as a tetramer (Morlot and Roux, 2013) that fluctuates between a monomer-tetramer equilibrium under physiological salt conditions (Binns et al., 1999; Hinshaw, 2000). These tetramers are capable of further self-assembly into rings or helices (Figures 3B,C) (Hinshaw and Schmid, 1995; Muhlberg et al., 1997; Binns et al., 1999).

Dynamin rings (or stacks of rings) eventuate under low salt conditions and no underlying template (Hinshaw and Schmid, 1995). In the presence of a template, e.g., liposomes, microtubules, or other suitable membrane templates, dynamin forms helices (Shpetner and Vallee, 1989; Stowell et al., 1999; Roux et al., 2010). The helical structure occurs due to interactions between the stalk dimers, which drive the assembly into the polymer of the expected size, as observed in the molecular dynamics of the assembly process (Faelber et al., 2011). The dimers polymerise in a curved alignment allowing the formation of the macromolecular helix (Faelber et al., 2011). The stalks form the core of the dynamin helix, with the GTPase domains facing outside and the PH domains on the inside bound to the membrane tube (Figure 3C) (Zhang and Hinshaw, 2001; Chen et al., 2004; Mears et al., 2007). Helix constriction, as seen in membrane fission in endocytosis, is believed to be the result of a conformation change at the flexible hinge of the BSE (Ford et al., 2011). This conformational change would also result in the PH domain inserting into the lipid bilayer, causing additional curvature stress on the membrane assisting in membrane fission (Ramachandran et al., 2009).

Dynamin self-assembly produces up to a 1000-fold increase in its GTPase activity (Anggono and Robinson, 2009). Of the isoforms, dynamin 2 has the highest intrinsic GTPase activity (3–10-fold higher than dynamin 1) and also a higher stimulated GTPase activity under self-assembly promoting conditions (Höning et al., 1997; Warnock et al., 1997). This oligomerization explains dynamin's ability to tubulate membrane bilayers under appropriate conditions by forming a continuous membrane coat around them (Takei et al., 1995; Sweitzer and Hinshaw, 1998; Roux et al., 2006) and its ability to associate with tubular templates which facilitate with its assembly (Shpetner and Vallee, 1989; Stowell et al., 1999; Marks et al., 2001; Roux et al., 2010). Dynamin polymerization in solution is favoured by binding to non-hydrolysable analogues of GTP, such as GTPγS, GDP·AlF${}_{4}^{-}$, or GMPPCP, while GTP hydrolysis favours disassembly of the dynamin oligomers and release of its subunits from the plasma membrane (Hinshaw and Schmid, 1995; Carr and Hinshaw, 1997; Marks et al., 2001; Danino et al., 2004). In the absence of a nucleotide, purified dynamin assembles into a helix of ~50 nm outer diameter with a helical pitch between 10 nm and 20 nm, surrounding a 20 nm radius membrane tubule (Sweitzer and Hinshaw, 1998; Takei et al., 1998; Chen et al., 2004; Danino et al., 2004). Dynamin assembly and hydrolysis of GTP is critical for completion of CME.

## Clathrin Mediated Endocytosis

**Figure 4:**
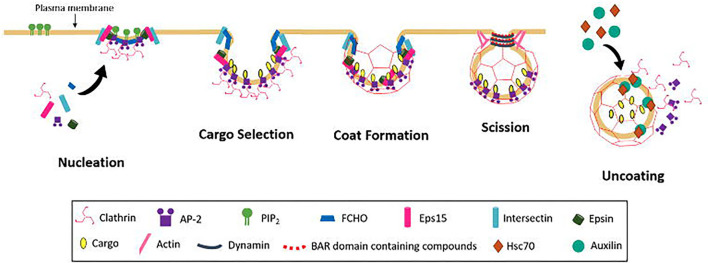
Schematic diagram of CCV formation occurring in the following steps; Nucleation, cargo selection, coat formation, vesicle scission, and uncoating.

Endocytosis spans a number mechanistically varied pathways including phagocytosis, micropinocytosis, caveolae-mediated endocytosis, and CME (McMahon and Boucrot, 2011; Kaksonen and Roux, 2018). CME is heavily implicated in nutrient uptake, signal transduction, synaptic vesicle recycling, maintenance of cell polarity, and antigen presentation (Mellman and Warren, 2000). CME can be broken up into five major stages and can be explained by examining the mechanism of cargo internalisation (Figure 4).

### Nucleation

The first step of CME is the nucleation of clathrin coated pits and vesicles (CCP and CCV), the exact mechanism of which remains to be elucidated. Key endocytic proteins include adaptor protein 2 (AP-2), FCH domain only (FCHO) proteins, epidermal growth factor receptors (EGFRs) and intersectins. All have binding domains for PI(4,5)P_2_, which is believed to act in the recruitment of endocytic proteins to the membrane (Höning et al., 2005; Reider et al., 2009; Stimpson et al., 2009; Henne et al., 2010; Taylor et al., 2011; Umasankar et al., 2012). It is still unclear if these components are all essential and which initiates the nucleation process. The AP-2 complex is known to be involved in the nucleation process for recruitment of clathrin, but whether the complex is necessary for initiation to occur is disputed (Motley et al., 2003; Godlee and Kaksonen, 2013). Depletion of AP-2 leads to a general block of transferrin (Tf) uptake (Godlee and Kaksonen, 2013), but this conflicts with other studies on the uptake of other major cargos, e.g., low-density lipoprotein receptor LDLR, EGFR and influenza (Motley et al., 2003). It may be that different cargo depend on different clathrin adaptors. Clathrin can be recruited to the membrane by other adaptors including adaptor protein 180 (AP180) and epsin, which have been found to recruit clathrin via synthetic lipid monolayer (Ford et al., 2001). These alternative adaptors may also work with AP-2 as some are found to bind to the AP-2 complex.

Depletion of FCHO reduced the lifetime of nucleation sites. CCPs still formed in the absence of FCHO, but were observed to abort, resulting in incomplete pit formation, and furthermore they were observed to grow faster in the presence of FHCO (Cocucci et al., 2012). FCHOs are also known to interact with epidermal growth factor receptor substrate 15 (eps15) and intersectin, both of which are important in progression of CCP formation (Benmerah et al., 1999; Koh et al., 2007; Pechstein et al., 2010). Epsin and intersectin, are believed to aid in the clustering of FCHO (Henne et al., 2010; Godlee and Kaksonen, 2013), additionally FCHO proteins and epsin, are often present selectively at the outer rim of the assembling coat and are proposed to have a role in stabilisation the curvature of the bilayer, via the F-Bar domain of FCHO (Tebar et al., 1996; Saffarian et al., 2009; Henne et al., 2010). FCHO proteins are necessary to stabilise the growing CCPs.

### Cargo Selection

CME generates the necessary vesicles to ensure transport of a wide variety of cargo across the plasma membrane. Protein components of the clathrin coat recruit cargo by binding to specific sites of different transmembrane cargo molecules, enriching the concentration of specific cargo at the site of the forming vesicle (Kaksonen and Roux, 2018). Recognition and internalisation of such a wide range of cargo requires the existence of many adaptor proteins. In CME, these adaptors are known as clathrin-associated sorting proteins (CLASPs). Clathrin and CLASPs binding allows the sorting proteins to become engrained in the polyhedral scaffold and engage in recognition of sorting signals to recruit cargo. CLAPSs are categorised by their structural identity as oligomeric or monomeric (Traub and Bonifacino, 2013).

AP-2 is the best understood cargo selective clathrin adaptor (Traub, 2009). AP-2 is a key player in CCP initiation, where it is able to bind to clathrin and PI(4,5)P_2_ (Cocucci et al., 2012). The AP-2 protein family comprises AP-1, AP-3, AP-4 and AP-5. AP-1 acts in essentially the same role as AP-2, but in CME at intracellular membranes, rather than the plasma membrane where AP-2 is observed. AP-3 has a role in the sorting of proteins to lysosomes (Hirst and Robinson, 1998). It is capable of acting with or without association with clathrin. AP-4 has no clathrin association, and the role of AP-5 is at this stage unclear (Robinson, 2015).

**Figure 5:**
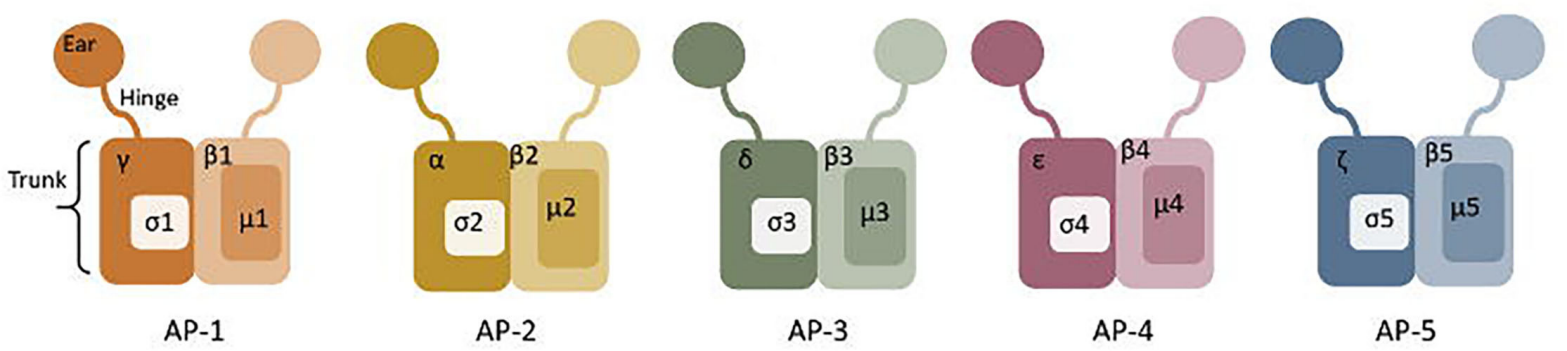
Structure of Adaptor complexes (AP) 1-5. The hinge, truck and ear regions are labelled on AP-1, and the subunits are labelled on each adaptor protein.

The AP complexes are each made up of four non-identical polypeptide chains arranged into a block-like shape, with two symmetrical appendages connected by a flexible hinge-like stalk (Figure 5) (Hirst and Robinson, 1998; Traub, 2009). AP-2 comprises an α subunit (100 kDa), β2 subunit (100 kDa), μ2 subunit (50 kDa), and σ2 subunit (17 kDa) (Scarmato and Kirchhausen, 1990; Collins et al., 2002; Traub, 2009). The two hinge appendages (α and β2 subunits) are involved in interaction with clathrin and other adaptors, while the central block-like core mediates sorting signal recognition and binding to PI(4,5)P_2_ (Traub, 2009).

In addition to the AP complexes, a wide range of dimeric and monomeric CLASPs have also been characterised. Some examples of dimeric sorting proteins include FCHO1 and eps15, and monomeric CLASPs include Low Density Lipoprotein Receptor Adaptor Protein 1 (LDLRAP1 or ARH), Disabled homologue (Dab), Numb, β-arrestin and epsin (Traub and Bonifacino, 2013). CLASPs identify and recruit cargo through the use of endocytic signals, typically linear amino acid motifs (Ohno et al., 1995; Olusanya et al., 2001; Höning et al., 2005; McMahon and Boucrot, 2011; Taylor et al., 2011; Traub and Bonifacino, 2013) and covalent modifications, such as ubiquitination (Sorkina et al., 2006) and phosphorylation (Santini et al., 2002; Edeling et al., 2006; Burtey et al., 2007; Marchese et al., 2008; Reider et al., 2009; Traub and Bonifacino, 2013).

### Coat Formation

Coat assembly occurs as clathrin triskelia begin to self-polymerise to a polyhedral cage around the cargo (Kaksonen and Roux, 2018), leading to a CCV that is still connected to the plasma membrane by a narrow “neck” (Young, 2007). As polymerisation occurs curvature effectors such as EPS15 and epsin are displaced to the edges of the forming vesicle (Tebar et al., 1996), to assist in the stabilisation of the coat (McMahon and Boucrot, 2011). There are two competing models of membrane bending to form the CCP proposed (Kaksonen and Roux, 2018); the constant curvature model (Kirchhausen, 2009; Saffarian et al., 2009; Cocucci et al., 2012) and the constant area model (Avinoam et al., 2015; Haucke and Kozlov, 2018).

The constant curvature model was originally proposed, where clathrin would polymerise directly onto a curved membrane, maturation occurring via an increase in the area of the clathrin coated membrane. The area increases as clathrin self assembles, while the curvature of the membrane remains constant throughout maturation of the CCV (Kirchhausen, 2009; Saffarian et al., 2009; Cocucci et al., 2012). Many *in vitro* findings are consistent with the constant curvature model as increased tension and bending rigidity blocked clathrin polymerisation (Saleem et al., 2015), however *in vivo* flat clathrin patches have repeatedly been observed (Heuser, 1980; Mettlen et al., 2010), leading to the proposal of the constant area mode.

The constant area model requires the formation of a clathrin lattice on a planar or nearly planar area of membrane prior to the initiation of membrane bending (Avinoam et al., 2015). Molecular rearrangements and exchange of clathrins results in the hexagon/pentagon curved coat (Kirchhausen, 1993; Avinoam et al., 2015), increasing the curvature while the area remains largely unchanged (Avinoam et al., 2015). The exchange of clathrin at the CCP is observed during both early and late stages of endocytosis (Kirchhausen et al., 2014) and is supported by the results that blocking the turnover of clathrin arrests CCP formation at all stages (Von Kleist et al., 2011). More recent studies have also supported this model showing curvature beginning after assembly of a flat clathrin lattice (Bucher et al., 2018; Scott et al., 2018). However, the number of clathrin bonds that would need to undergo modification to form a pentagon from a hexagon paired with the affinity of these bonds renders this an energetically costly process (Kirchhausen, 1993; Nossal, 2001). It is likely that the process occurs by a combination of these models, as both models have been simultaneously observed, showing curvature prior to clathrin polymerisation, and curvature after clathrin polymerisation (Haucke and Kozlov, 2018; Scott et al., 2018).

The integration of accessory proteins such as epsin or clathrin assembly lymphoid myeloid leukaemia protein (CALM) within the lattice may assist in the curvature (Ford et al., 2002; McMahon and Boucrot, 2011; Miller et al., 2015; Kaksonen and Roux, 2018). Actin filaments are also able to contribute to membrane curvature, via localised polymerisation at the membrane and coupling with the clathrin coat to force the bending of the membrane (Carlsson and Bayly, 2014; Kaksonen and Roux, 2018).

### Scission

**Figure 6:**
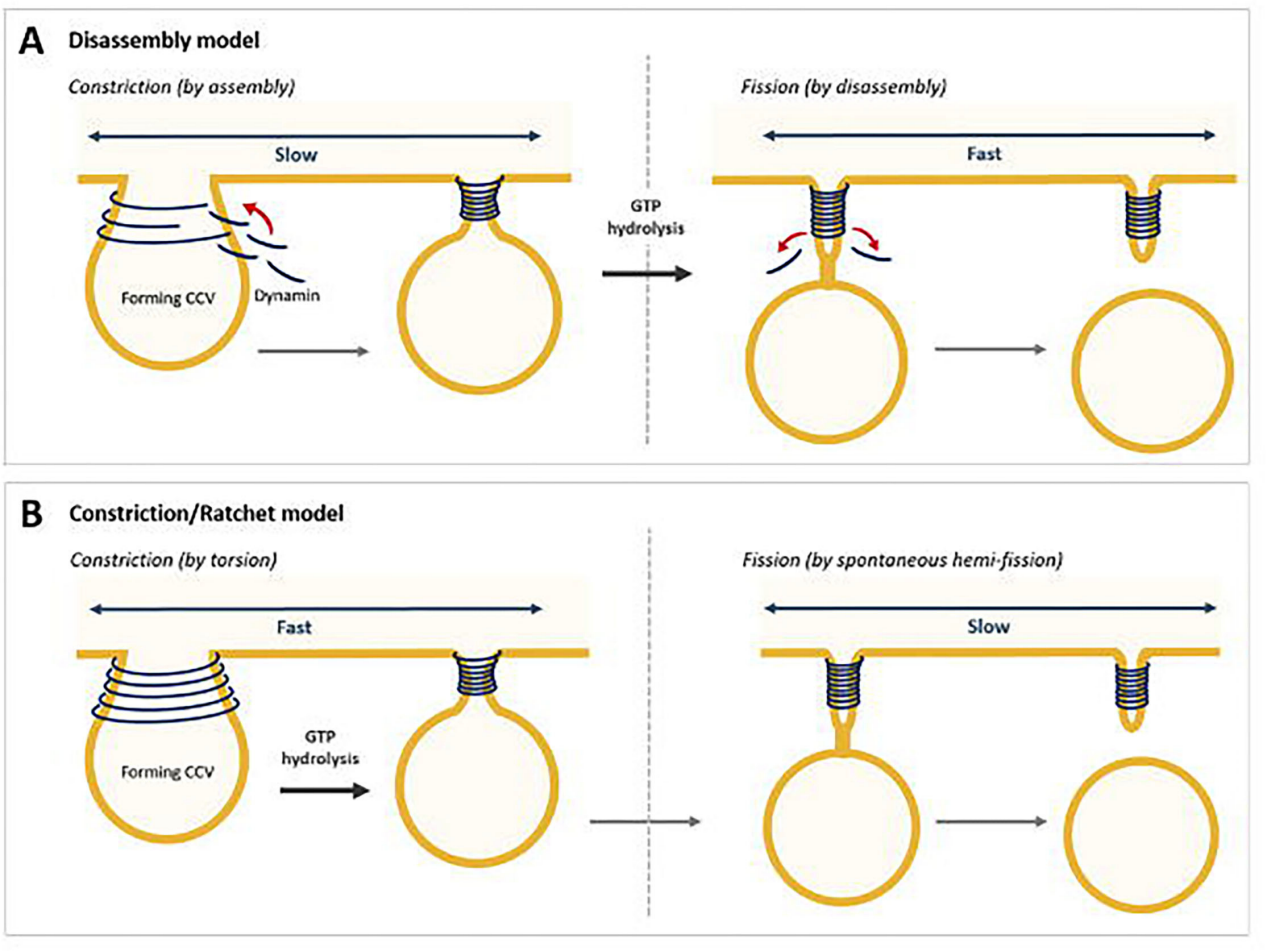
Two models for dynamin-mediated membrane fission. **(A)** Two-stage fission model, where constriction is mediated by assembly and fission by disassembly. **(B)** The constriction/ratchet model, where constriction is achieved by active sliding of the helical turns and induce fission (Chen et al., 2020).

Scission of the narrow neck that connects the almost fully formed membrane bound vesicle allows completion of cargo trafficking. This is mediated by dynamin as a helical collar around the neck of the formed vesicle (Kaksonen and Roux, 2018). Dynamin recruitment to the vesicle is believed to occur by BAR domain containing proteins, e.g., amphiphysin, endophilin, sorting nexin 9 (SNX9) via binding at the PRD (McMahon and Boucrot, 2011). GTP hydrolysis modulates the helical structure of dynamin, (Sweitzer and Hinshaw, 1998) and membrane tension facilitates the constriction that reduces the radius of the membrane in its helix (Stowell et al., 1999; Roux et al., 2006). There are two current models of dynamin fission step (Antonny et al., 2016); two-stage dynamin-catalysed fission model (disassembly model) and the constrictase/ratchet model (Figure 6).

The two-stage dynamin-catalysed fission model suggests that in the first stage, assembled dynamin in a specific nucleotide-driven conformation adopts a super-constricted state enabling the formation of a hemi-fission intermediate (Bashkirov et al., 2008; Pucadyil and Schmid, 2008; Boucrot et al., 2012). This stage likely corresponds to the GDP + P_i_ transition state, when GTPase domains across adjacent rungs form their highest affinity interactions (Chappie et al., 2011). Release of P_i_ from the GTP-bound state in the second stage would then release the scaffold, as seen by negative-stain electron microscopy, allowing for the hemi-fission intermediate to proceed to complete fission (Figure 6A) (Stowell et al., 1999; Danino et al., 2004; Mattila et al., 2015).

The constrictase/ratchet model suggests dynamin functions as a motor. The energy obtained from GTP hydrolysis would be spent in mechanical work to slide adjacent turns of the dynamin helix (Figure 6B). In this model, GTPase domains, which binds dynamin through direct interactions, could act as molecular motors, and by the dimerizations-power strokes-dissociations cycles powered by GTP hydrolysis, would trigger relative sliding of the helical turn, leading to constriction and twisting of the helix. Fission results from membrane destabilisation arising when the constricted ring disassembles (Sweitzer and Hinshaw, 1998; Praefcke and McMahon, 2004; Ferguson and De Camilli, 2012; Antonny et al., 2016). Studies have shown that GTP binding induces trans-dimerization of dynamin GTPase domains via an interface across the nucleotide-binding site (Chappie et al., 2011). BSE senses the nucleotide loading status of the GTPase domain resulting in an open conformation in the presence of GTP and a 70° rotation to a closed state in the absence of nucleotide. Potentially this acts as a power stroke for dynamin during constriction and twisting (Chappie et al., 2009, 2010, 2011; Ford et al., 2011).

### Uncoating

The final step in CME involves the release of endocytic proteins by uncoating allowing these materials to be recycled and the vesicle to fuse with its target membrane for cargo trafficking (Kaksonen and Roux, 2018). Auxilin binds within the clathrin lattice at the points of overlapping ankles, which lie under the tripod of the C-terminal of the adjacent triskelion (Fotin et al., 2004b; Kaksonen and Roux, 2018) recruiting heat shock cognate (Hsc70) to a critical point of interaction of the clathrin lattice (Schlossman et al., 1984; Umeda et al., 2000; Fotin et al., 2004b). Auxilin binding is believed to distort the clathrin lattice (Xing et al., 2010), allowing Hsc70 recruitment and binding at the QLMLT sequence under the tripod of the central vertex (Rapoport et al., 2009), increasing distortion and strain at the point where three ankles cross over each other under the tripod (Rapoport et al., 2009; Xing et al., 2010). This Hsc70 mediated strain between clathrin triskelion is one of the two possible mechanisms for uncoating understood at this stage. The other model suggests the collision resulting from the recruitment of Hsc70 produced a localised pressure in the disrupted lattice (Xing et al., 2010) to mediate uncoating (Sousa et al., 2016; Kaksonen and Roux, 2018).

## Neurological Diseases Related to SVR

### Synaptic Vesicle Recycling

Neurotransmission between nerve cells is at the core of intracellular communication in the sensory and nervous systems. Non-peptide neurotransmitters are released from small, specialised round organelles called synaptic vesicles (SVs), involved in the signalling across synapses (Wu et al., 2014; Milosevic, 2018). SVs are essential in preserving key neuronal properties, but there are a limited number of SVs in the brain due to the small size of presynaptic terminal. Thus, synapses reply on controlled recycling of SV (SVR) membranes and proteins. SVR is fundamental to synaptic transmission (Lu et al., 2009; Chanaday et al., 2019).

At pre-synapses SVs are organised into four “pools”; the readily releasable pool (RRP), recycling pool, reserve pool and resting pool (Chanaday et al., 2019). The RRP consist of SVs that are physically in contact with the plasma membrane and are immediately available for fusion. Upon the arrival of electrical membrane potential (action potential), Ca^2+^ channel opening is induced, and Ca^2+^ concentration elevation stimulates fusion of these SVs in the RRP. The RRP requires constant replenishment, which occurs either by recruitment of new SVs from the reserve pool, or the rapid reuse of fused SVs (Holderith et al., 2012; Guo et al., 2015).

SVR involves vesicle exocytosis from the RRP, which is followed by retrieval via endocytosis (Lu et al., 2009; Denker and Rizzoli, 2010; Kaeser and Regehr, 2017) to ensure a constant supply of neurotransmitter filled SVs. Following endocytosis, new SVs required rapid refilling with neurotransmitters and prepared for fusion (Blakely and Edwards, 2012; Farsi et al., 2017). Vacuolar H^+^ ATPase (vATPase) and vesicular neurotransmitter transporters are the main components involved in the filling of new SVs. vATPase acts by forming an electrochemical gradient across the membrane, which allows the transporters to move the neurotransmitters into the SVs. The transporters also determine the type of neurotransmitters content in the SV. Recycling of these key components is believed to be coupled with SVR (Blakely and Edwards, 2012; Farsi et al., 2017; Chanaday et al., 2019).

Investigations in a range of different systems, indicated an essential role of clathrin and suggested CME is one of the major mechanism for SVR (Heuser and Reese, 1973; Maycox et al., 1992; Morgan et al., 1999, 2000, 2001; Granseth et al., 2006; Heerssen et al., 2008; Kasprowicz et al., 2008). CME at the synapses is a specialised form of the membrane trafficking pathway that occurs in all cells, occurring via the same five steps (nucleation, cargo selection, coat formation, scission, uncoating) as detailed above. However, the rate of endocytosis during SVR differs vastly from endocytosis at the plasma membrane (Morris and Schmid, 1995), and unlike the heterogeneous vesicles observed at the plasma membrane CME at the synapses results in extremely uniform vesicles (Milosevic, 2018). Synaptic vesicles are internalised in seconds following neurotransmission, and are able to be re-loaded with neurotransmitters within 1–2 min; in contrast, the half time of internalisation of transferrin (Tf) at the plasma membrane is 3–5 min (Morris and Schmid, 1995). Clathrin independent mechanisms of SVR will not be discussed in further detail here, for a more complete overview refer to recent reviews in this area (Cremona and De Camilli, 1997; Soykan et al., 2016; Watanabe and Boucrot, 2017; Chanaday et al., 2019; Casamento and Boucrot, 2020; Renard and Boucrot, 2021).

SVR originally was thought to comprise only “kiss and run” and CME. Kiss and run occurs quickly (<1–2 s) compared to CME (10–30 s) (Chanaday et al., 2019). In addition, kiss and run does not require clathrin-associated proteins, and instead acts through SV briefly contacting the plasma membrane via a small fusion pore, which allows the release of small molecules. The SVs do not completely flatten to the membrane and are then able to be quickly recycled (Fesce et al., 1994; Gandhi and Stevens, 2003; Wightman and Haynes, 2004; Zhang et al., 2009; Wen et al., 2017). SVR is now know to encompass activity dependent bulk endocytosis (ADBE) (Holt et al., 2003; Wu and Wu, 2007), clathrin independent fast endocytosis (CIFE) (Delvendahl et al., 2016), fast endophilin mediated endocytosis (FEME) (Boucrot et al., 2015; Casamento and Boucrot, 2020; Renard and Boucrot, 2021), ultrafast endocytosis (UFE) (Clayton and Cousin, 2009; Clayton et al., 2009; Holderith et al., 2012; Watanabe and Boucrot, 2017; Gan and Watanabe, 2018).

ADBE occurs in response to longer bursts of intense activity and retrieves large areas of membrane within 1–2 s via a clathrin independent mechanism (Clayton et al., 2008; Clayton and Cousin, 2009; Kononenko and Haucke, 2015; Gan and Watanabe, 2018; Chanaday et al., 2019). Large intracellular endosomes are formed by inducing actin-driven membrane invaginations at sites where synaptic vesicle cargo is clusters. These endosomes average 150 nm, but can reach up to 500 nm in size and non-specifically retrieve all molecules within that region of the membrane (Clayton and Cousin, 2009; Kononenko and Haucke, 2015; Nicholson-Fish et al., 2015). In mammalian, invertebrate and amphibian synapses, ADBE is triggered by high stimuli and increased Ca^2+^ concentrations. Increased Ca^2+^ levels in nerve terminals activate calcineurin, which dephosphorylates and activates many endocytic proteins, including synapnin-1 and dynamin-1 (Cheung and Cousin, 2013). In weakly stimulated neurons ADBE is inhibited by the phosphorylation of these endocytic proteins by kinases [including Cyclin dependent kinase 5 (Cdk5) and glycogen synthase kinase 3 β (GSK3β)] (Evans and Cousin, 2007; Smillie et al., 2013). Some studies have indicated ADBE can occur independently of dynamin (Raimondi et al., 2011; Wu et al., 2014).

UFE can occur as fast as 50 ms after exocytosis (Watanabe et al., 2013a,b; Zhou et al., 2014; Delvendahl et al., 2016). UFE studies in *C. elegans* (neuromuscular junctions) and in mouse (hippocampal synapses) and have shown sites of UFE as the edges of the active zone, and is predominant as physiological temperatures (Watanabe et al., 2014; Soykan et al., 2017). UFE results in larger vesicles (80 nm) and does not require clathrin. Although not requiring clathrin, many of the proteins required for CME are also involved in ultrafast endocytosis. Synaptojanin 1, endophilin A and dynamin 1 are involved in vesicle budding and scission, while actin is also essential (Watanabe et al., 2014; Watanabe and Boucrot, 2017; Chanaday et al., 2019). There are also reports of CIFE, mediated by dynamin and actin which additionally shares many features of UFE (Delvendahl et al., 2016).

FEME is also clathrin independent but requires many CME proteins, notably regulation by endophilin. FEME occurs within 1–10 s and is triggered on activation of specific receptors, including G-protein coupled receptors, interleukin-2 receptor (IL2R) and some receptor tyrosine kinases (Boucrot et al., 2015). This pathway occurs through a similar progression of steps as CME and has been suggested to function in the post-synaptic SVR (Boucrot et al., 2015; Watanabe and Boucrot, 2017; Casamento and Boucrot, 2020).

All the known components of CME, including clathrin and dynamin, are expressed in higher levels (10–50-fold) in neuronal cells, possibly allowing the accelerated rate of CME observed, by increasing the number of coated pits and efficiency of assembly (Morris and Schmid, 1995). Neuron-specific inserts are observed in both clathrin light chains (Brodsky et al., 1991), and also in the β-subunit of AP-2 complex, along with the neuron specific dynamin 1 (Robinson et al., 1994), and the α-subunit of AP-2 (Robinson, 1992; Morris and Schmid, 1995). There are also neuron specific endocytic components, not generally observed in CME at the plasma membrane such as neuron specific assembly protein AP-3, which is ca 4 times more efficient at stimulating clathrin assembly (Lindner and Ungewickell, 1992; Morris and Schmid, 1995).

In addition to its role in CME recycling of synapses, clathrin also mediates the steps involved in filling SVs with neurotransmitters, as sorting of neurotransmitter transporters requires clathrin and adaptor proteins (AP-1, AP-2, and AP-3) (Blakely and Edwards, 2012; Silm et al., 2019). Each SVs must contain a proper set of neurotransmitter transporters to ensure the SV is filled with the correct neurotransmitter content (Chanaday et al., 2019). Additionally clathrin may play a role in determining timely acidification, and therefore neurotransmitter loading. Acidification of SVs requires uncoating of the clathrin cage, suggesting removal of this coat ensures the timing of neurotransmitter loading occurs as soon as the SV is reformed (Farsi et al., 2018).

The efficient and effective recycling of SVs is critical for proper function of sensory and nervous systems, and synaptic physiology (Milosevic, 2018). Various neurological conditions have proposed reliance on SVR and possibly CME, such as Parkinson's (Inoshita and Imai, 2015; Vidyadhara et al., 2019), Alzheimer's (Rafii and Aisen, 2009; Wu and Yao, 2009; Palmer, 2011; Alsaqati et al., 2018), epilepsy (Chin et al., 1995; Di Paolo et al., 2002; Kim et al., 2002), Huntington's disease (McAdam et al., 2020), and schizophrenia (Schubert et al., 2012).

### CME Defects Contributing to Disease

It is important to recognise that the aetiology of neuronal disease is typically complex. It is thus difficult to assign defects in a single process or protein as being the cause of the disease, but in the exemplars (note that this is not intended as a comprehensive or exhaustive list of endocytosis related human diseases) shown below, a defect in endocytosis has been reported.

### Alzheimer's Disease

Alzheimer's disease, affects >37 million people globally, is a progressive neurological disorder that affects the daily lives of sufferers through cognitive impairment and memory loss (Rafii and Aisen, 2009). Early symptoms include issues with short term memory, spatial orientation, attention, confusion and changes in mood and personality (Palmer, 2011).

The amyloid precursor protein (APP), a type of glycoprotein, is produced by the neuron and has several roles in the development and function of the neuron (Mattson, 1997). APP can undergo cleavage by either non-amyloidogenic processing (α-secretase and γ-secretase) or amyloidogenic processing (β-secretase and γ-secretase). The latter of these results in production of amyloid-β peptide (Aβ) (Alsaqati et al., 2018), which along with neurofibrillary tangles, are central to the pathogenesis of Alzheimer's disease (Rafii and Aisen, 2009). It is suggested that endocytosis and intracellular sorting determines the processing of APP, and that amyloidogenic processing occurs within endosomes, the production of Aβ is therefore dependent on internalisation of APP via CME (Alsaqati et al., 2018). One of the most notable abnormalities in AD is changes in the endocytic process (Milosevic, 2018). An increase in endocytosis with age has been reported, leading to increased internalisation of APP and subsequently production of Aβ, potentially explaining why age is the most important risk factor of AD (Milosevic, 2018). Further, it has been demonstrated that the proteins involved in CME are upregulated with ageing, including dynamin and clathrin (Alsaqati et al., 2018).

### Huntington's Disease

Huntington's Disease (HD) is a genetic neurodegenerative disease with main symptoms including dementia, ataxia and chorea. The disease is caused by a mutation in the huntingtin (HTT) gene, resulting in dysfunction of the huntingtin protein (Htt) leading to specific degeneration of spatial medium spiny neuron, causing psychiatric, motor and cognitive symptoms in addition to disturbances in the mitochondrial electron transport chain (Zeviani and Carelli, 2005; Bates et al., 2015). Genetic studies have shown a link between Htt and CME (Harjes and Wanker, 2003; Zeviani and Carelli, 2005; Singh et al., 2017; McAdam et al., 2020). The aggregation of the mutated Htt protein has been shown to cause inhibition of CME, inhibiting the internalisation of receptors and recycling of SVs affecting neuronal function (Yu et al., 2014). Htt has been observed to interact with a range of proteins implicated in SVR, specifically clathrin-mediated SVR. The Htt aggregate sequesters HSC70, an essential protein in CME, specifically in the uncoating process, rendering it unable to act in this process and inhibiting endocytosis (Yu et al., 2014). Htt has additionally been shown to interact directly with AP-2, and is involved in recruitment of AP-2 to the membrane. A loss of this docking function, due to mutation of the proteins results in reduced recruitment of AP-2 and impairment of CME.

### Charcot Marie Tooth Disease and Centronuclear Myopathy

Charcot Marie Tooth (CMT) disease is one of the most common inherited neurological diseases affecting 1 in 2,500 people. Symptoms include muscle weakness and foot ulcers, often causing infection, due to the characteristic impaired sensory and motor neuronal functions (Szigeti and Lupski, 2009; Singh et al., 2017). CMT has been linked to mutations of intracellular trafficking related genes, specifically in the PHD of dynamin 2 (Durieux et al., 2010).

Similarly, autosomal-dominant Centronuclear myopathy (CNM), an inherited neuromuscular disorder, has been speculated to result from mutations in dynamin 2 (Bitoun et al., 2005; Jungbluth et al., 2008). CNM is characterised by delayed motor milestones, muscle weakness, progressive muscle wasting, cognitive impairment, extraocular muscle palsy and frequently limited eye movement (Bitoun et al., 2005; Jungbluth et al., 2008; Sidiropoulos et al., 2012; Singh et al., 2017). Currently there is no effective treatment available, however DNM2 (dynamin gene) knockdowns have been shown to improve muscle mass and restore muscle structure (Buono et al., 2018). This led to investigation of dynamin 2 knockdown mediated by antisense oligonucleotide (ASOs), the lead candidate (developed by DynaCure) is currently undergoing human trials. Mice studies showed the ASO knockdown resulted in a reduction of dynamin 2 levels in muscle tissue and prevented myopathy from developing, while ASO treatment of affected mice lead to the correction of muscle defects (Tasfaout et al., 2017).

### Parkinson's Disease

Parkinson's Disease (PD) is an age-dependent neurodegenerative disorder associated with tremors, slow movement, sleep disturbances, cognitive difficulties and depression (Inoshita and Imai, 2015; Singh et al., 2017). These symptoms occur as a result of dopamine depletion and loss of dopamine neurons in the mid brain. Although the specific molecular mechanism that causes the neurodegeneration is unclear, recent developments have indicated that many of the causative PD genes are involved in regulation of vesicular trafficking, including SVR (Inoshita and Imai, 2015). For example, mutations of an E3 ubiquitin protein ligase, Parkin, results in early onset autosomal recessive PD. Association between Parkin and endophilin, the endocytotic protein that binds and directs dynamin to the necks of CCVs, indicated the possible role of Parkin in regulation SVR and pathogenesis of PD. Late onset autosomal dominant PD is believed to be associated with a mutation in leucine-rich repeat kinase 2 (LRRK2). LRRK2 also interacts with endophilin, and is involved in regulation of SV dynamics, with additional links to dynamin (Inoshita and Imai, 2015; Singh et al., 2017).

### Epilepsy

Affecting ~50 million people worldwide, epilepsy is a central nervous system disorder with spontaneous seizures as the main symptom (Li et al., 2015; Vannini et al., 2020). In lesional epilepsy, seizures develop in response to brain damage (Pitkänen and Immonen, 2014), while in non-lesional epilepsy they are the result of altered synaptic function (Farisello et al., 2013; Vannini et al., 2020). These seizures are caused by high frequency, synchronous and uncontrolled synaptic transmission in the brain (Li et al., 2015; Vannini et al., 2020). SVR plays a clear role in this abnormal synaptic transmission by maintaining the neurotransmission of the central synapses (Li et al., 2015).

Due to their role in SVR, CME and its components have implications in altered synaptic function. In addition there is significant evidence of dynamins role in epileptic seizures (Anggono et al., 2006; Appenzeller et al., 2014). Mutations in dynamin 1 have been observed to cause epileptic symptoms, while in both animal models and human epilepsy dynamin 1 is upregulated and may contribute to the development of seizures, with dynamin inhibition, by the small molecule inhibitor Dynasore, exhibiting anti-epileptic effects (Appenzeller et al., 2014; Li et al., 2015; Vannini et al., 2020). Moreover the clinically used levetiracetam (Keppra^TM^) is believed to act directly at synaptic vessel protein 2 (SV2A) (Lynch et al., 2004; Klein et al., 2020; Contreras-García et al., 2021).

### Schizophrenia

Affecting ~0.5–1.0% of the worldwide population, schizophrenia is a debilitating neuropsychiatric disorder characterised by a breakdown in thinking, attributed to defects in synaptic transmission. Several schizophrenia-susceptibility genes have been identified, including dysbindin (dystrobrecin-binding protein 1) (Chen et al., 2008). This gene is known to effect neurotransmission, resulting in the cognitive dysfunctions associated with the disorder and is also believed to effect hippocampal dopamine levels. Dysbindin is involved in processes that are closely related CME (Chen et al., 2008; Schubert et al., 2012). Additionally, key aspects of the neuropathology, such as white matter changes, synaptic dysfunction and abnormal neurodevelopment are all influenced by CME and clathrin dependent processes (Schubert et al., 2012). The phenothiazine-based antipsychotic drugs, such as chlorpromazine, are now known to strongly inhibit CME, via inhibition of dynamin (Daniel et al., 2015). First and second generation antipsychotics are also known to interact with β-arrestin, another clathrin interacting protein involved in CME (Masri et al., 2008; Schubert et al., 2012; Singh et al., 2017).

## Inhibitors of Clathrin and Dynamin

Currently there are no available treatments to cure neurodegenerative diseases, with treatments aiming to support patients, manage symptoms and halt disease progression (Durães et al., 2018; Overcoming Gaps in the Treatment of Neurodegenerative Disease, 2020). Drug development in this area has focused primarily on postsynaptic targets and G-protein coupled receptors, leaving the investigation of endocytic machinery as potential drug targets an underexplored, but promising avenue.

The inhibition of CME through genetic and chemical means has been used to explore and reveal the molecular components required for, and also the consequences of, blocking CME. Studies into the inhibition of CME have highlighted its potential medical importance, e.g., in the entry of bacterial and viral pathogens into cells (Dutta and Donaldson, 2012), psychiatric disorders (schizophrenia and bipolar disorder) (Schubert et al., 2012) and neurodegenerative diseases [e.g., Parkinson's (Vidyadhara et al., 2019) and Alzheimer's disease] (Alsaqati et al., 2018). Viral pathogens, such as the influenza virus (Yang et al., 2011), Ebola virus (Bhattacharyya et al., 2010), African Swine fever virus (ASFV) (Hernaez and Alonso, 2010), Simian Haemorrhagic fever virus (Caì et al., 2015), HIV (Aggarwal et al., 2017), and Semliki Forest virus (SFV) (Doxsey et al., 1987) have been found to hijack CME to enter cells (Harper et al., 2013). Also known to enter via CME are some bacterial pathogens and yeast including *Staphylococcus aureus* (Veiga et al., 2010) and uropathogenic *Escherichia coli* (Eto et al., 2008), and Toxins such as Anthrax toxin (from *Bacillus anthracis*) (Abrami et al., 2010) and diphtheria toxin (from *Corynebacterium diphtheria*) (Skretting et al., 1999; Harper et al., 2013). As CME is one of the major endocytic pathways involved in SVR, investigation of mechanisms of inhibition of CME may provide understanding of the synapse function along with the potential of therapeutic targets, improving knowledge of neurodegenerative processes. CME machinery is a promising target for development of new therapeutics for neurodegenerative disorders (Konno et al., 2012; Schubert et al., 2012; Oliver and Reddy, 2019), specifically this section will focus on inhibitors of dynamin and clathrin, along with general CME inhibitors.

### CME Inhibition–Generic Approaches to CME Inhibition

Classical inhibition of CME includes environmental stimuli, which act as general cellular perturbants, such as cytosolic acidification, potassium depletion, and hypertonic treatment. These methods, while gleaning information into the endocytic process, also effect many non-clathrin mediated pathways as well as many non-endocytic processes resulting in unknown global effects on the cells (Dutta and Donaldson, 2012).

Hypertonic sucrose (0.4–0.5 M) results in the dispersion of regular clathrin lattices and the formation of microcages (Carpentier et al., 1989; Ivanov, 2008), but has additionally be found to interfere with all three major internalisation pathways and cause cell shrinkage (Ivanov, 2008; Dutta and Donaldson, 2012). Potassium ion (K^+^) depletion is another classical method of CME inhibition. Once K^+^ reached a critical level in cells, the number of clathrin coated pits is reduced by ~80%, and CME rate is decreased by 70–95% (Carpentier et al., 1989; Ivanov, 2008). This is believed to be cause by aggregation of clathrin in the cytoplasm, and although is thought to be more selective to clathrin dependent endocytic pathways (Ivanov, 2008). A range of side effects are observed including reduced protein and DNA synthesis (Dutta and Donaldson, 2012). Similarity, cytosol acidification is observed to cause a range of side effects, however its mode of action against CME differs, the clathrin coated vesicles are “frozen” at the cell surface when the pH is <6.5 (Cosson et al., 1989; Dutta and Donaldson, 2012).

There have also been genetic approaches employed to inhibit CME by altering the expression of specific protein, such as using the expression of mutants or siRNA-mediated depletion of proteins. Expression of a mutant form of dynamin (K44A, patterned after a temperature sensitive mutant in *Drosophila*) has been shown to inhibit CME, however this also resulted in an increased the rate of clathrin-independent fluid endocytosis (Van der Bliek et al., 1993; Damke et al., 1994). Additional studies show expression of the CTD of clathrin, the carboxyl-terminal clathrin binding domain of AP180 (AP180C) or a truncated from of Eps15 (lacking the epsin homology domain) are all able to inhibit CME (Benmerah et al., 1998; Liu et al., 1998; Zhao et al., 2001). Unfortunately overexpression of proteins (wild-type and mutants) is believed to lead to a range of possible indirect off target effects (Dutta and Donaldson, 2012).

Another approach is the knockdown of proteins by siRNA (e.g., siRNA of clathrin has been shown to block the formation of CCPs) (Hinrichsen et al., 2003), however the considerable time (3–7 days) required to deplete cells, and the cell adaptions and altered gene expression possible in this time are significant drawbacks to this approach (Motley et al., 2003; Dutta and Donaldson, 2012). These drawbacks mean it is difficult to ensure CME is the only impacted pathway. The “knock-sideways” approach was developed to circumvent some of these drawbacks, and involved the translocation of clathrin (to the mitochondria) to deplete cells of clathrin, although there are concerns of the diversion of other proteins in the process, resulting in off-target effects (Robinson et al., 2010; Dutta and Donaldson, 2012).

Although genetic approaches are useful for studies into the understanding of mechanisms of CME, these also resulted in a range of side effects and are not methods that can be readily translated to a therapeutic use.

### CME Inhibition–Small Molecules CME Inhibitors

Firstly, a note on the use of small molecule inhibitors. All small molecules have potential off-target effects, even those published as specific inhibitors (Hopkins, 2008; Hafner et al., 2019; Oña and Bouso, 2020). To ameliorate these potential effects and to strengthen evidence associated with the observed phenotypes, that are ascribed to the inhibition of the target protein, it is strongly recommended (a) that more than one chemical scaffold that inhibits the target protein is used in the biochemical study (it is unlikely that two (or more) chemical scaffolds will display the same off target effects); and (b) inactive control compounds using as close a structural analogue as possible is used (chemically similar but with no *in vitro* target efficacy). Combined these approaches can significantly improved target validation. A listing of the known dynamin and clathrin inhibitors with inhibition data (where published) is provided in Table 2.

**Table 2:** IC_50_ of compounds discussed in this review.

**Compound**	**Clathrin IC_**50**_ (μM)**	**Dynamin 1 IC_**50**_ (μM)**	**Dynamin 2 IC_**50**_ (μM)**	**CME IC_**50**_ (μM)**	**References**
**Unknown target**					
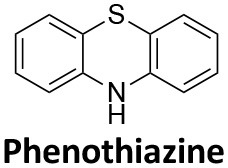	-	> 300	No activity	No activity	Daniel et al., 2015
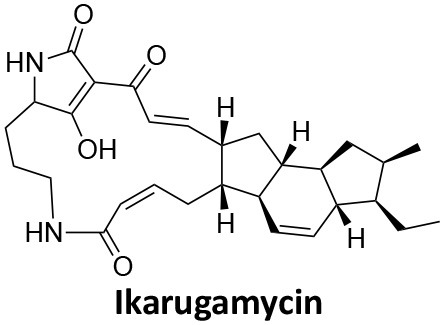	-	-	-	2.7 ± 0.3 Tf uptake in H1299 cells	Elkin et al., 2016
**Clathrin inhibitors**					
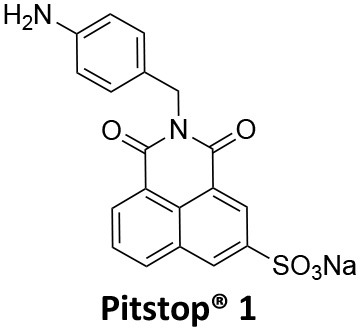	18 ELISA ^a^	-	-	-	Von Kleist et al., 2011
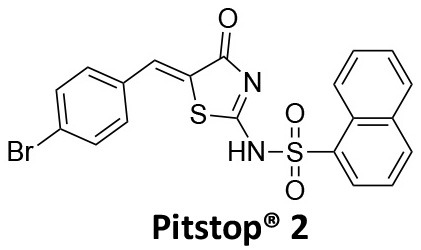	12 ELISA^a^	-	-	9.7 ± 1.5 Tf uptake in U2OS cells	Von Kleist et al., 2011
**Dynamin inhibitors**					
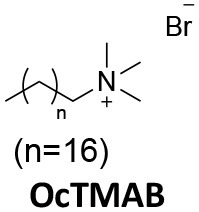	-	1.9 ± 0.24 20 nM^b^ 0.92 ± 0.13 7 nM^b^	4.4 ± 2.4 20 nM^c^	16.0 ± 4.2 EGF-A4888 in COS-7 cells	Hill et al., 2004; Quan et al., 2007; Joshi et al., 2010
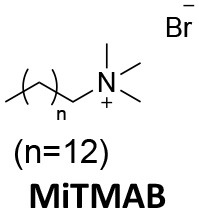	-	3.1 ± 0.2 20 nM^b^ 2.26 ± 0.53 7 nM^b^ 24.1 ± 9.4 20 nM^d^	8.4 ± 5.8 20 nM^c^	20.9 ± 3.2 EGF-A4888 in COS-7 cells	Hill et al., 2004; Quan et al., 2007; Otomo et al., 2008; Joshi et al., 2010
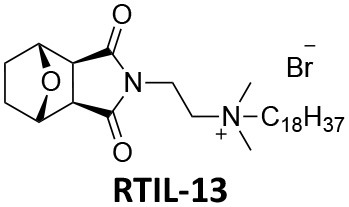	-	2.3 ± 0.3 20 nM^b^	-	-	Zhang et al., 2008
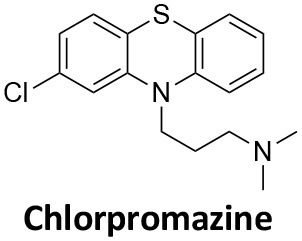	-	6.8 ± 1.5 50 nM^b^ 47.2 ± 23.1 20 nM^d^	4.1 ± 2.5 50 nM^f^	17.4 ± 2.4 Tf uptake in U2OS cells	Otomo et al., 2008; Daniel et al., 2015
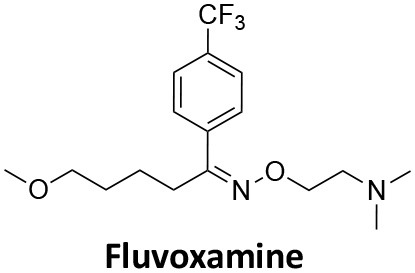	-	14.7 ± 1.6 20 nM^d^	-	-	Otomo et al., 2008
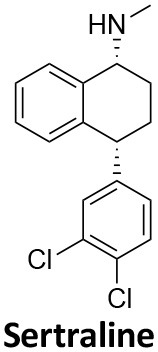	-	7.3 ± 1.0 20 nM^d^	3.7 ± 1.3 20 nM^e^	-	Otomo et al., 2008
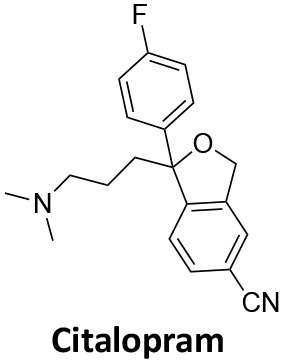	-	> 100 20 nM^d^	-	-	Otomo et al., 2008; Takahashi et al., 2010
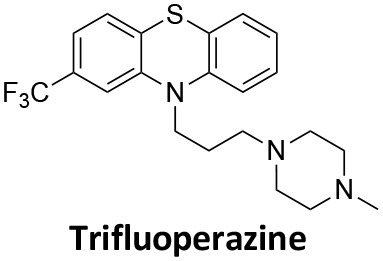	-	2.6 ± 0.7 50 nM^b^	2.5 50 nM^f^	10.4 ± 1.7 Tf uptake in U2OS cells	Daniel et al., 2015
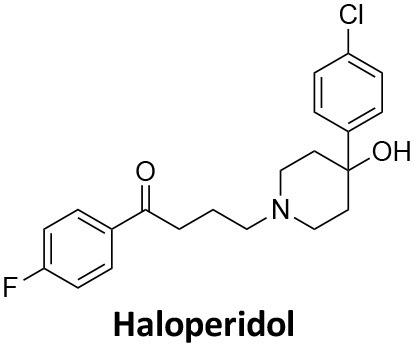	-	19.0 ± 2.2 50 nM^b^	6.5 50 nM^f^	54.5 ± 23 Tf uptake in U2OS cells	Daniel et al., 2015
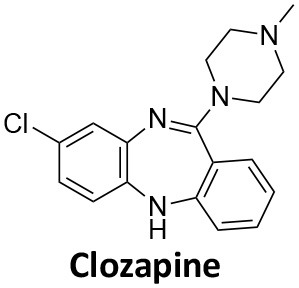	-	28.2 ± 1.2 50 nM^b^	5.3 50 nM^f^	85.3 ± 14 Tf uptake in U2OS cells	Daniel et al., 2015
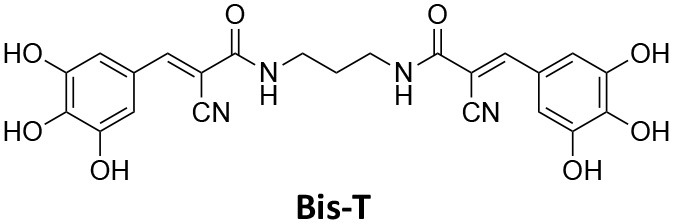	-	1.7 ± 0.2 200 nM^b^	-	-	Hill et al., 2005
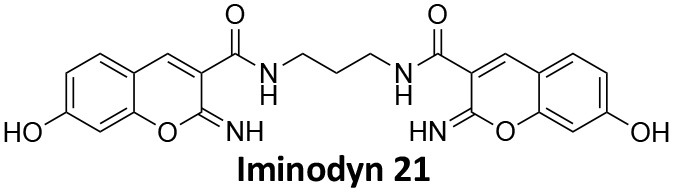	-	17.3 ± 1.0 20 nM ^b^	16.7 ± 36 69 nM^c^	No activity Tf uptake in U2OS cells	Hill et al., 2010
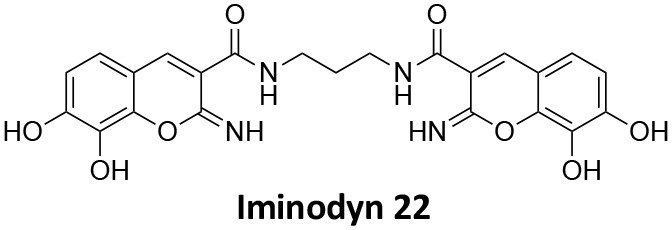	-	0.45 ± 0.05 20 nM^b^	0.39 ± 0.15 69 nM^c^	10.7 ± 4.5 Tf uptake in U2OS cells	Hill et al., 2010
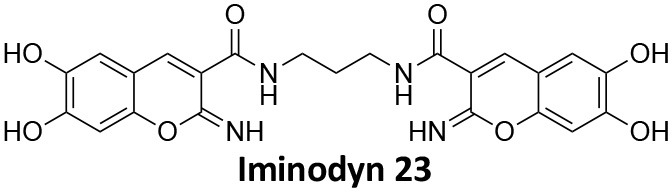	-	0.26 ± 0.08 20 nM^b^	0.29 ± 0.11 69 nM^c^	74.6 ± 8.8 Tf uptake in U2OS cells	Hill et al., 2010
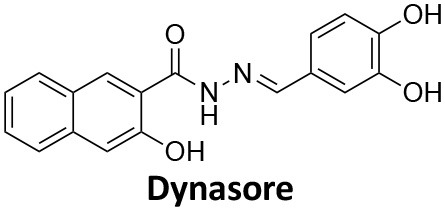	-	12.4 ± 1.5 10–20 nM^b^	-	34.7 ± 5.1 Alexa 594-Tf uptake in U2OS cells	Macia et al., 2006; Mccluskey et al., 2013
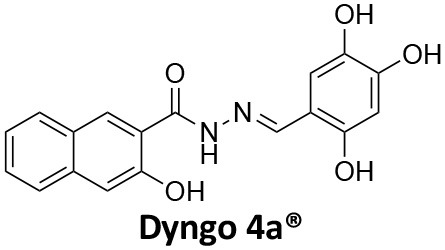	-	0.38 ± 0.05 10–20 nM^b^ 1.1 ± 0.2 50 nM^g^	2.3 ± 0.2 50 nM^f^	5.7 ± 1.0 Alexa 594-Tf uptake in U2OS cells	Mccluskey et al., 2013
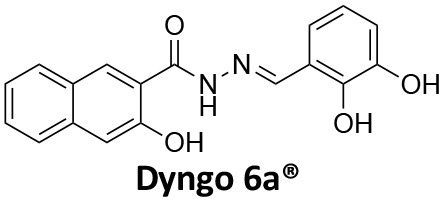	-	3.2 ± 0.3 10–20 nM^b^	-	5.8 ± 0.8 Alexa 594-Tf uptake in U2OS cells	Mccluskey et al., 2013
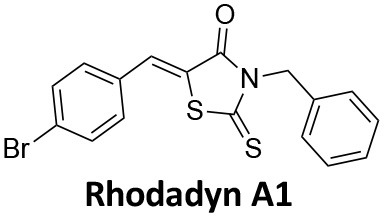	-	134 ± 21 20 nM^b^	-	-	Robertson et al., 2012
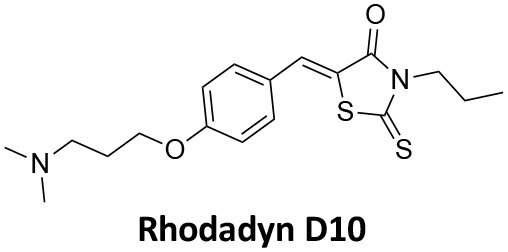	-	4.5 ± 0.8 20 nM^b^	-	5.9 ± 1.0 Tf uptake in U2OS cells	Robertson et al., 2012
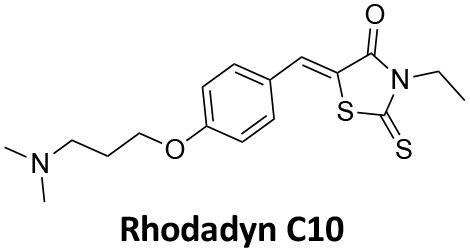	-	7.1 ± 1.9 20 nM^b^	-	7.0 ± 2.2 Tf uptake in U2OS cells	Robertson et al., 2012
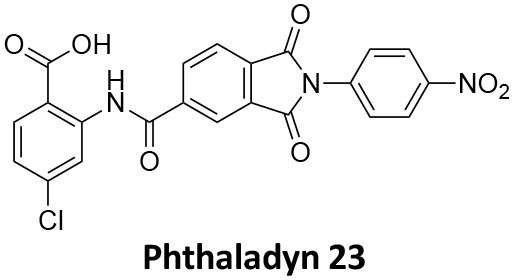	-	17.4 ± 5.8 20 nM^b^	63 ± 33 20 nM^c^	No activity Tf uptake in U2OS cells	Odell et al., 2010
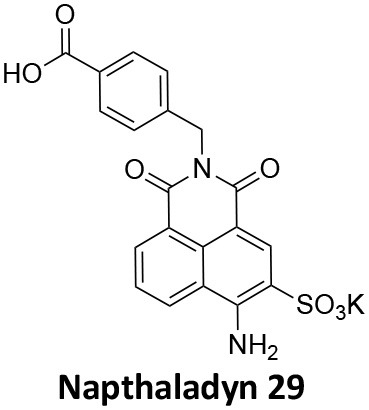	-	18.5 ± 1.7 20–30 nM^b^	-	66 Tf uptake in U2OS cells	Abdel-Hamid et al., 2015
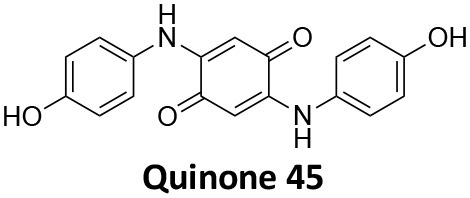	-	11.1 ± 3.6 50 nM^b^	23.6 50 nM^f^	36 ± 16 Alexa 594-Tf uptake in U2OS cells	Macgregor et al., 2014b
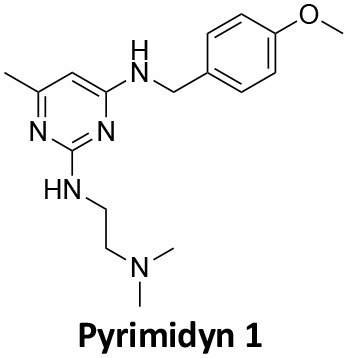	-	35.3 ± 7.1 20 nM^b^	> 100 50 nM^f^	211 ± 37.1 Alexa 488-conjugated EGF uptake in COS-7 cells	McGeachie et al., 2013
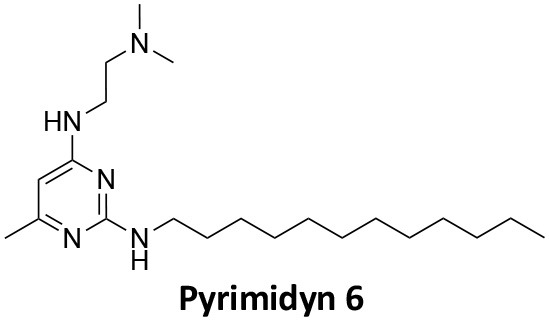	-	1.4 ± 0.2 20 nM^b^	12.4 ± 4.9 50 nM^f^	19.6 ± 3.5 Alexa 488-conjugated EGF uptake in COS-7 cells	McGeachie et al., 2013
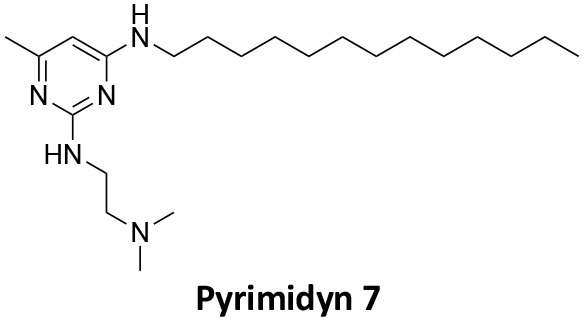	-	1.1 ± 0.05 20 nM^b^	12.1 ± 8.0 50 nM^f^	12.1 ± 2.1 Alexa 488-conjugated EGF uptake in COS-7 cells	McGeachie et al., 2013
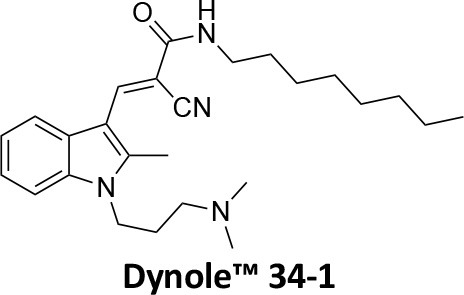	-	3.33 ± 0.75 20 nM^b^	-	10.8 ± 1.4 Tf uptake in U2OS cells	Hill et al., 2009
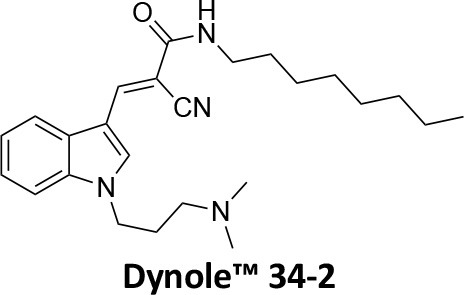	-	1.30 ± 0.30 20 nM^b^	-	5.0 ± 0.9 Tf uptake in U2OS cell	Hill et al., 2009
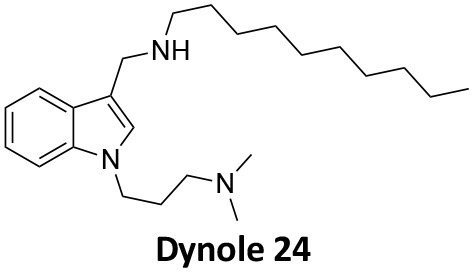	-	0.56 ± 0.09 20 nM^b^ 1.7 ± 0.3 50 nM^b^	5.4 ± 7.5 50 nM^f^	1.9 ± 0.3 Tf uptake in U2OS cells	Gordon et al., 2013
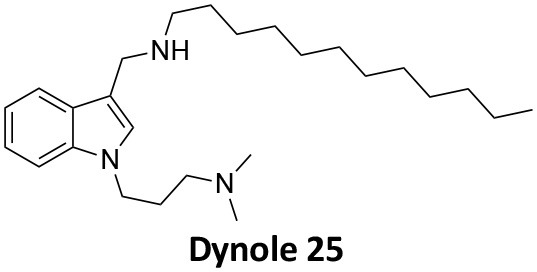	-	0.76 ± 0.05 20 nM^b^	-	2.8 Tf uptake in U2OS cells	Gordon et al., 2013

*Compounds discussed with no reported IC_50_'s are not included. MDC, chloroquine and PAO all have an unknown target and no reported IC_50_'s. Bolinaquinone is known to target clathrin, but at this time there is no known IC_50_'s, similarly BPs (etidronate, clodronate, and tiludronate) target dynamin but currently have no reported IC_50_'s. Studies of ES9 and ES9-17 report EC_50_'s so have not been included. Dynamin 1 and 2 IC_50_'s were determined via Malachite Green GTPase assay, with variations in the concentration of dynamin as notes. CME IC_50_'s generally determined by Texas Red-Tf Uptake assay*.

a *Inhibition of amphiphysin association with CTD*,

b *Endogenous dynamin 1 purified from sheep brain*,

c *Recombinant Dyn2-His_6_ from insect cells (Sf9)*,

d *Recombinant Dyn1-His_6_ from E. coli*,

e *Recombinant Dyn2-His_6_ from E. coli*,

f *Recombinant Dyn2-His_6_ from insect cells (Sf21)*,

g *Recombinant Dyn1-His_6_ from insect cells (Sf21)*.

**Figure 7:**
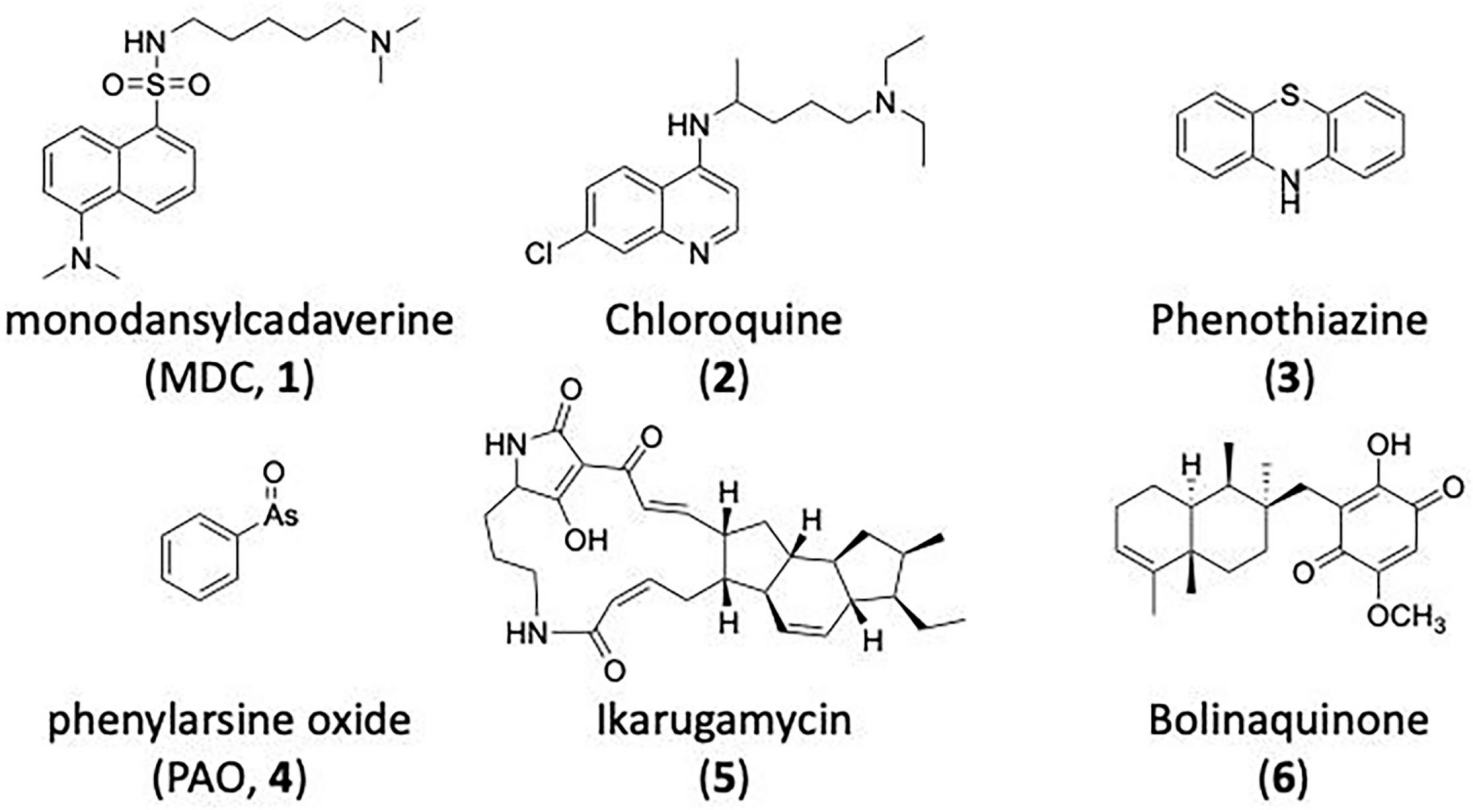
Structures of small molecules CME inhibitors; monodansylcadaverine (MDC, **1**), chloroquine (**2**), phenothiazine (**3**), Phenylarsine oxide (PAO, **4**), Ikarugamycin (**5**), and bolinaquinone (**6**).

Monodansylcadaverine (MDC, **1**) (Figure 7) is known to stabilise CCV to uncoating and inhibit CME. MDC (**1**) displays a range of side effects believed to be due to its inhibitory action against enzymes of the transglutaminase family, which may affect the organisation and dynamics of the actin cytoskeleton. Some studies have also observed inhibition of macropinocytosis and phagocytosis by MDC (**1**) (Schlegel et al., 1982; Kang et al., 1995; Singh et al., 2003; Mishra and Murphy, 2004). Similar to MDC (**1**), chloroquine (**2**) and phenothiazine (**3**) (Figure 7) are hydrophobic amines which inhibit CME via affecting the function and formation of CCVs (Dutta and Donaldson, 2012). The modes of action of MDC (**1**) and chloroquine (**2**) are largely unknown; however, phenothiazine (**3**) has been shown to block dynamin (Daniel et al., 2015; Chew et al., 2020). These compounds have been observed to exhibit off target effects and non-specificity to clathrin dependent endocytic pathways (Chen et al., 2009; Dutta and Donaldson, 2012).

Phenylarsine oxide (PAO, **4**) inhibits CME at 1–20 μM, via an unknown mechanism (Figure 7). PAO (**4**) exhibits a range of serious side effects, which result in the disorganisation of action cytoskeleton, along with non-specific as inhibition of macropinocytosis and phagocytosis (Francesco Retta et al., 1996; Gerhard et al., 2003; Singh et al., 2003).

Ikarugamycin (**5**) (Figure 7) displays broad-spectrum antibiotic and antiprotozoal activity (Jomon et al., 1972). It was subsequently found to be cytotoxic (IC_50_ = 0.22 μM, HL-60 cells) (Popescu et al., 2011). Ikarugamycin (**5**) reversibly inhibits transferrin endocytosis in H1299 cells (IC_50_ of 2.7 ± 0.3 μM) (Hasumi et al., 1992; Elkin et al., 2016). Comparable activity is conserved across multiple human cell lines (H1299, HCC366, H1437, ARPE-19, and HBEC3KT), and Ikarugamycin (**5**) showed no inhibition of albumin uptake [via Caveolae-Mediated Endocytosis (CavME)] or CD44 and CD59 uptake [via clathrin independent endocytosis (CIE)] over a 1–4 μM concentration range (Elkin et al., 2016). However, despite the promising indication of CME selectivity of Ikarugamycin (**5**), cytotoxicity is observed at concentration below CME activity and the mode of action is currently unknown (Elkin et al., 2016).

## Small Molecule Clathrin Inhibitors

### Small Molecule Chemical Probes

The clinical use of small molecules, drugs, is a major goal in therapeutic interventions. However, prior to attaining this outcome, it is often essential to validate the target-disease nexus and the therapeutic suitability of the proposed approaches. In this regard, the development of probe molecule is often the first step in this process. These probe molecules may be the ultimate drug candidate, but it is more common that these molecules are used in the initial target validation steps. They are most often not regarded as drugs. During this interrogation of a pathway it is generally considered prudent that multiple probes (and inactive controls as described above) be used in confirming (or not) the expected phenotype from inhibiting the target protein. The current palette of inhibitors targeting clathrin and dynamin are best described as early-stage probe molecules.

### Bolinaquinone

The marine sesquiterpene hydroxyquinone, bolinaquinone (**6**) (Figure 7) (De Guzman et al., 1998), was identified as a possible clathrin inhibitor in a proteomics experiment. The selective pull-down of clathrin after attachment to agarose beads at the methoxy position identified (**6**) as a clathrin inhibitor (Margarucci et al., 2011). Initial studies showed *in vitro* cytotoxicity against a human colon cell line and broad spectrum anti-inflammatory effect (Lucas et al., 2003; Petronzi et al., 2011). Albumin internalisation studies on THP1 (human acute lymphoma) additionally validate the CME activity of (**6**), which was found to be non-cytotoxic at concentrations <100 μm (Margarucci et al., 2011). A computational docking study investigated the possible CTD binding site of bolinaquinone (**6**), leading to the identification of a proposed new fifth site between blades 5 and 6.

### ES9-17

**Figure 8:**
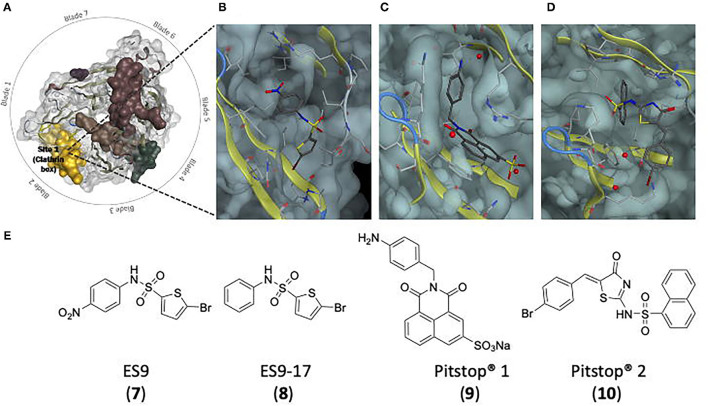
Binding site and structures of small molecule clathrin inhibitors. **(A)** Top view of the CTD, showing Site 1 (in yellow) the binding site of ES9 (**7**), ES9-17 (**8**), Pitstop® 1 (**9**), and Pitstop® 2 (**10**) (Ter Haar et al., 1998). **(B)** ES9 in site 1 of the CTD (Dejonghe et al., 2019). **(C)** Pitstop® 1 in site 1 of the CTD. **(D)** Pitstop® 2 in site 1 of the CTD (Von Kleist et al., 2011). **(E)** Chemical structures of ES9 (**7**), ES9-17 (**8**), Pitstop® 1 (**9**), and Pitstop® 2 (**10**).

Endosidin 9 (ES9, **7**) (Figure 8E) is a known endocytic inhibitor and inhibits the uptake of a tracer dye (FM4-64) in *Arabidopsis* root epidermal cells (IC_50_ = 5 μM). As ES9 (**7**) is a protonophone, inhibition may be the result of cytosolic acidification, however it is believed the activity may be structure driven as CME activity is still observed at high apoplectic pH (Dejonghe et al., 2019). ES9 (**7**) led to the synthesis of several related analogues including a non-protonophore lead; ES9-17 (**8**). *In silico* modelling studies led to the proposal that ES9 (**7**) and ES9-17 (**8**) bind in site 1 of the CTD (Figure 8A). This binding site was confirmed by the determination of the CTD co-crystal with ES9 (**7**), indicating it is likely ES9-17 (**8**) is binding at site 1 also (Figure 8B). ES9-17 (**8**) effectively inhibits CME in *Arabidopsis*, and to date is the only known small molecule inhibitor allowing reversible inhibition of CME in *Arabidopsis*. ES9-17 (**8**) was also found to inhibit transferrin uptake in human cells (HeLa cells) after treatment with 30 μM, however at this stage there is no reported IC_50_ (Dejonghe et al., 2019).

### Pitstops®

The Pitstop® series of compounds were identified via screening of a library of ~17,000 small molecules, leading to the synthesis of two structurally distinct leads. Subsequent focused library development and screening afforded Pitstop® 1 (**9**) and Pitstop® 2 (**10**) (Von Kleist et al., 2011; Robertson et al., 2016) (Figure 8E). These compounds have been found to inhibit the CTD and formation of clathrin coated pits, which in turn inhibited the CME entry of HIV and also interfered with SVR (Von Kleist et al., 2011). Although showing inhibition of transferrin uptake Pitstop® 1 (**9**) (IC_50_ = 18 μm, CME) possess limited cellular uptake, while Pitstop® 2 (**10**) exhibits cell permeability and inhibits transferrin uptake into HeLa cells (IC_50_ = 12–15 μm) (Smith et al., 2013). X-ray co-crystals of the Pitstop®–CTD complex revealed that although structurally different they both bind at Site-1 between blades 1 and 2 lying off-set relative to each other in a hydrophobic cavity comprising I52, I62, I80, I93, L82, and F91 (Figures 8A,C,D) (Von Kleist et al., 2011).

There has been some speculation about the specificity and mode of action of Pitstop® 2 (**10**). Previous results from knockout studies have observed the ability of CME to function with any one site functional (Lemmon and Traub, 2012). This has raised the question of how inhibition at the one known binding site of Pitstop® 2 (**10**) results in CME inhibition. Additionally, it has been demonstrated with various point mutation on the CTD that Pitstop® 2's inhibitory action was unhindered. Notable studies with a C+ mutant [which altered the key residues involved in the clathrin box site (site 1)] found Pitstop® 2 (**10**) activity to be conserved. It is thus possible that Pitstop® 2 (**10**) is able to bind at all sites on the CTD, which would explain the inhibition of CME (Elferink, 1979; Lemmon and Traub, 2012; Willox and Royle, 2012), or there is a second site of Pitstop® 2 (**10**) action. However, molecular docking studies performed with the altered sites alongside the existing Pitstop® 2/CTD co-crystal postulate that these altered amino acids are not involved in binding at all, and thus would not be sufficient to prevent Pitstop® 2 (**10**) inhibition but may in fact result in increased binding affinity (Robertson et al., 2016). Another study suggested the non-specificity of Pitstop® 2 (**10**) due to the observed inhibition of MHCI (major histocompatibility complex I) internalisation into HeLa cells, believed to occur via a clathrin-independent endocytosis (CIE) (Dutta et al., 2012; Willox et al., 2014). However, AP-2 μ2 and CHC knockdown studies also resulted in reduced entry to cells, confirming the MHCI pathway is clathrin/AP-2 dependent (Stahlschmidt et al., 2014). The specificity is further reinforced when observing Shiga toxin entry, a Clathrin independent process, of which Pitstop® 2 (**10**) provides no inhibition (Von Kleist et al., 2011; Dutta et al., 2012).

### Peptide Inhibitors: Wbox 2

**Figure 9:**
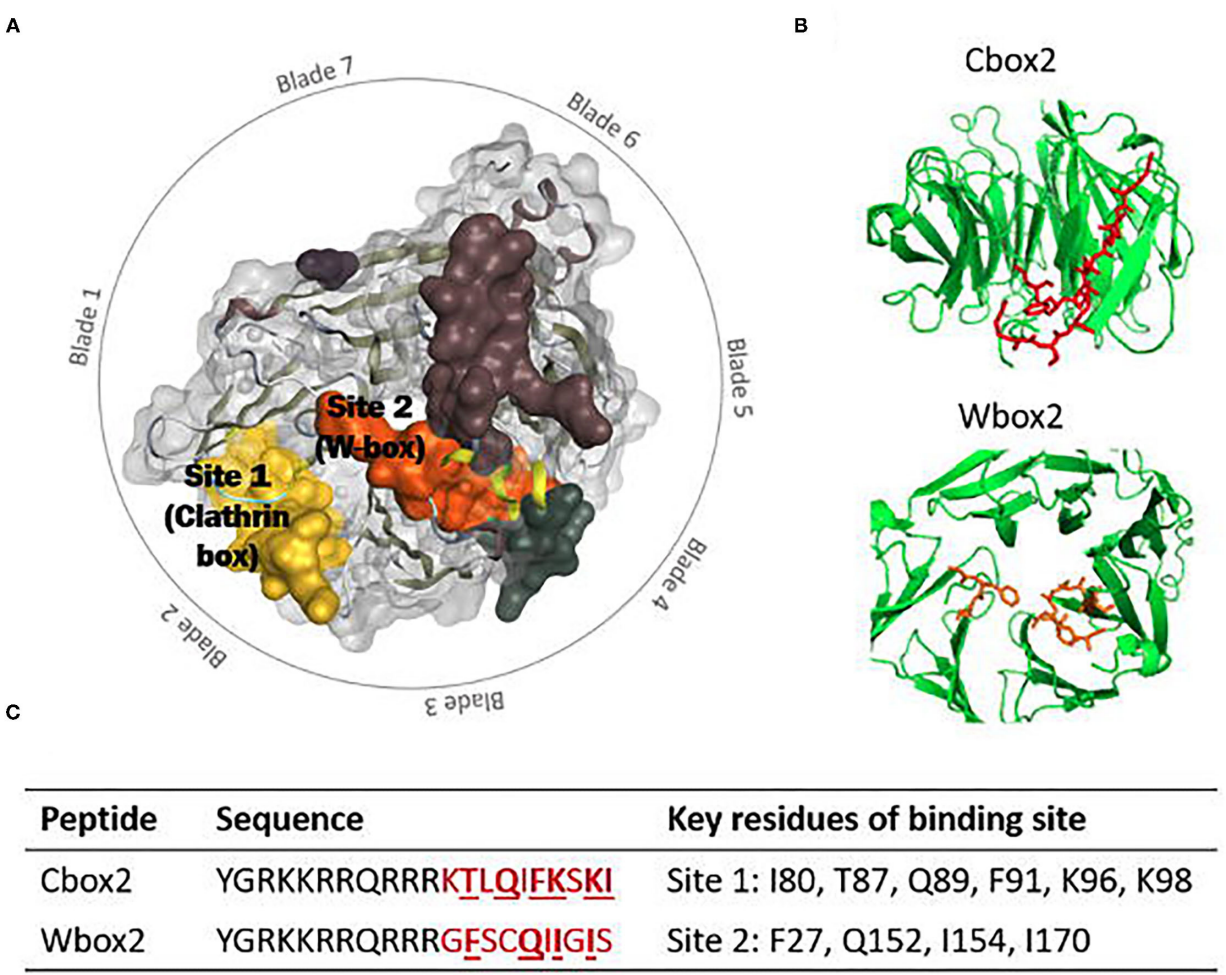
**(A)** Top view of the CTD, showing the binding sites of interest; Site 1 (clathrin box) in yellow and Site 2 (W-box) in orange (Ter Haar et al., 1998). **(B)** Amino acid sequences of Cbox2 (red) and Wbox2 (orange) shown on the CTD (Chen et al., 2020). **(C)** Peptide sequence of Cbox2 and Wbox2, TAT (transactivator of transcription) sequence in black and CTD derived amino acids in red. Bold and underlined are the key resides (corresponding to the key residues of Site 1 and 2) (Chen et al., 2020).

Overexpression of the CTD results in CME inhibition, shown using a CTD and distal leg (TDD) construct, which resulted in decreased transferrin uptake and additionally showed no significant inhibition of CIE (CD44 and CD59 uptake) (Chen et al., 2020). Overexpression of the TDD inhibited initiation and stabilisation of CCPs and blocked late-stage maturation of formed CCPs by interfering with the clathrin-AP-2 and clathrin-SNX9 interactions. The overexpressed terminal domain (TDD) is not incorporated into the clathrin/AP-2 coat, but rather binds AP-2 and SNX9, sequestering these proteins from normal CME functions, corresponding to an increase in the observation of AP-2 deficient CCP formation (Chen et al., 2020). This led to the design of a series of peptides encoding the key residues of each binding site (Chen et al., 2020). Two of these, Cbox2 and Wbox2, substantially and reversibly inhibited CME. Of the peptides designed, Cbox2 and Wbox2 were the only that encompassed the entire bonding motif, by taking into account discontinuous residues on adjacent blades (Figure 9). Of the peptides, Wbox2 showed the greatest inhibition (IC_50_ ~ 3 μM, Tf uptake in ARPE19/HPV16 cells), which was attributed to AP-2 and SNX9 binding following pulldown studies (Chen et al., 2020). However, Wbox2 was non-specific and found to inhibit clathrin independent endocytosis (CD44 and CD59 uptake) proposed to be due to either the upregulation of CIE during CTD overexpression or that SNX9 is required for CIE, and inhibition by Wbox2 sequesters it from CIE (Chen et al., 2020).

## Small Molecule Dynamin Inhibitors

### Long Chain Ammonium Salts

**Figure 10:**
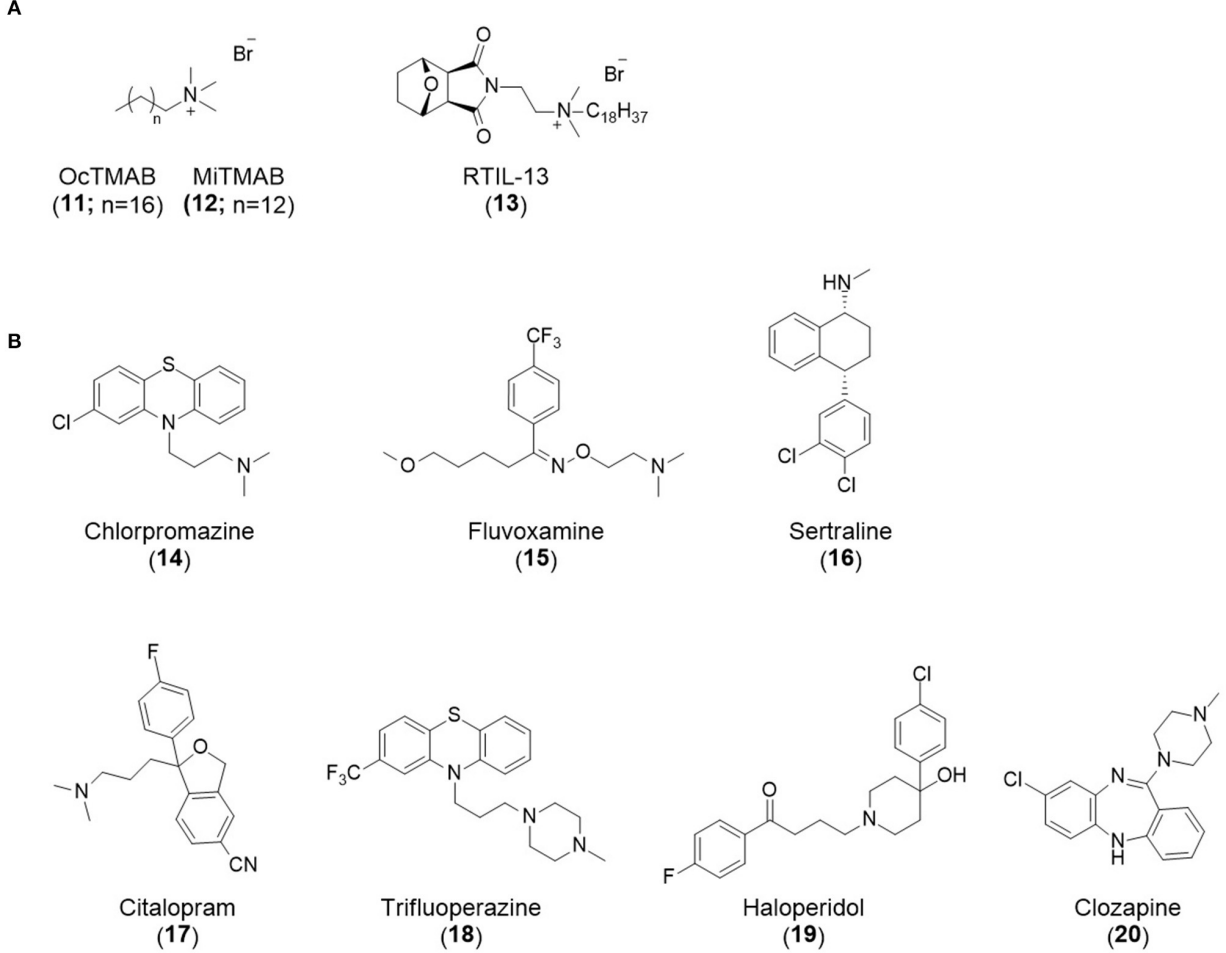
Structures of, **(A)** long chain ammonium salt dynamin inhibitors OcTMAB (**11**), MiTMAB (**12**) and RTIL-13 (**13**); and **(B)** anti-psychotics and Selective Seretonin Reuptake as dynamin inhibitors; chlorpromazine (**14**), fluvoxamine (**15**), sertraline (**16**), citalopram (**17**), trifluoperazine (**18**), haloperidol (**19**), and clozapine (**20**).

A primary screen of lipid like compounds resulted in the first class of compounds to be described as specific dynamin inhibitors (Hill et al., 2004). Originally long chain primary amines, it was predicted the hydrophobic molecular may potentially inhibit dynamin through interaction with the PH domain (Hill et al., 2004). The corresponding ammonium salts, octadecyl trimethyl ammonium bromide (OcTMAB, **11**) and myristyl trimethyl ammonium bromide (MiTMAB, **12**) (Figure 10A) were found to be the most potent with dynamin 1 IC_50_ values of 1.9 ± 0.24 μM and 3.1 ± 0.2 μM, respectively (Hill et al., 2004; Quan et al., 2007). Studies found these compounds disrupted the interaction of dynamin 1 with lipids, affecting the stimulation of GTPase activity (Quan et al., 2007). MiTMAB (**12**) was found to compete with PS binding to the PH domain of dynamin, as inhibition was not observed when using a recombinant dynamin 1 lacking the PH domain (dynamin 1-ΔPH). Additionally a sedimentation assay revealed MiTMAB (**12**) did not affect the self-assembly of dynamin (Quan et al., 2007). Cell based assays confirmed the inhibitory activity, specifically internalisation of transferrin and epidermal growth factor (EFG) into HeLa, Her14, A431, and COS-7 cells, mediated by dynamin 2 along with SVE inhibition mediated by dynamin as observed via styryl dye uptake measurements and synaptic vesicle depletion measurements (by electron microscopy) (Quan et al., 2007; Hill et al., 2010). These compounds also resulted in a significant reduction in the proliferation and viability of cancer cells, across a diverse panel of cell lines (Hopkins, 2008; Robinson et al., 2010).

### Room-Temperature Ionic Liquids (RTIL)

While developing new RTILs for the solubilisation of the dynamin PH domain for dynamic combination chemistry (DCC) assembly reactions, screening for potential dynamin inhibitors found RTIL-13 [4-(*N,N*-dimethyl-*N*-octadecyl-*N*-ethyl)-4-aza-10-oxatricyclo[5.2.1]decane-3,5-dione bromide, **13**], with an IC_50_ of 2.3 ± 0.3 μM (Figure 10A). The presence of an alkyl ammonium group revealed the structural similarity to MiTMAB (**12**) and led to the proposal of a similar mode of action, however this is currently not confirmed (Zhang et al., 2008; Eschenburg and Reubold, 2018).

### Psychotropic Drugs

Chlorpromazine (**14**) (Figure 10B), used to treat schizophrenia, inhibits CME originally thought to be due to inhibition of the clathirn-AP-2 complex (Wang et al., 1993). However, chlorpromazine's CME inhibition (**14**) occurs via dynamin inhibition (Daniel et al., 2015). The surfactant nature of MiTMAB (**12**) (a cationic surfactant at high concentrations) is shared with some pharmacologically active cationic amphiphilic compounds. Paired with the identification of chlorpromazine (**14**) as a CME inhibitor, this led to investigation of the inhibitory activity of a panel of selective serotonin reuptake inhibitors (SSRIs) and antidepressants against dynamin GTPase. The IC_50_ values of SSRI's were compared to the value obtained for MiTMAB (**12**) in this study, which gave an IC_50_ of 24.1 ± 9.4 μM (Otomo et al., 2008). The value was significantly less potent than the previously reported 3.1 ± 0.2 μM (Hill et al., 2004; Quan et al., 2007), a discrepancy believed to be observed due to the use of Dyn-His_6_ GTPase (from *E. coli*) in this study (Otomo et al., 2008) compared to native dynamin 1 (from sheep brain) (Hill et al., 2004; Quan et al., 2007). Two SSRI's displayed inhibitory effects greater that MiTMAB (**12**) [IC_50_ 24.1 ± 9.4 μM, against Dyn-His_6_ GTPase (from *E. coli*)]; fluvoxamine (**15**) (IC_50_ 14.7 ± 1.6 μM) and sertraline (**16**) (IC_50_ 7.3 ± 1.0 μM) (Otomo et al., 2008) (Figure 10B). Sertraline (**16**), along with chlorpromazine (**14**), blocked transferrin and cholera toxin subunit (CTB) endocytosis in HeLa cells. The related SSRI, citalopram (**17**), had no effect on endocytosis (Figure 10B) (Takahashi et al., 2010). The clinical efficacy of SSRI's means that these compounds are normally dosed at concentrations <100 time that required for *in vitro* CME inhibition. Caution is thus required even in using these SSRI's as probes to examine dynamin (and CME) inhibition. Given the potency differential, this would feasible only if the resultant phenotype was confirmed through the use of other dynamin inhibitors and the appropriate inactive control compounds. Realistically, though, the use of SSRI's to interrogate dynamin and CME is not encouraged.

The dynamin GTPase activity of a range of anti-psychotic drugs (APDs), both typical and atypical, were investigated. Typical APDs include chlorpromazine (**14**) and other phenothiazine derived compounds. All nine clinically used phenothiazine compounds were observed to inhibit dynamin 1 with the most potent being trifluoperazine (**18**) with an IC_50_ of 2.6 ± 0.7 μM. These compounds additionally inhibited dynamin 2 with similar IC_50_ values. Atypical APDs represent a more structurally diverse class of compounds of which five were investigated. Of these haloperidol (**19**) and clozapine (**20**) (Figure 10B), displayed improved potency for dynamin 2 (IC_50_ = 6.5 μM and 5.3 μM, respectively), over dynamin 1 (IC_50_ = 19.0 ± 2.2 μM and 28.2 ± 1.2 μM, respectively) with the remaining showing no activity (Daniel et al., 2015). Many of these compounds are highly non-specific, with activity against dopamine receptors (Ban, 2007) and phagocytosis (Elferink, 1979) among others, indicating multiple off target effects.

### Bis-Ts and Iminodyns

**Figure 11:**
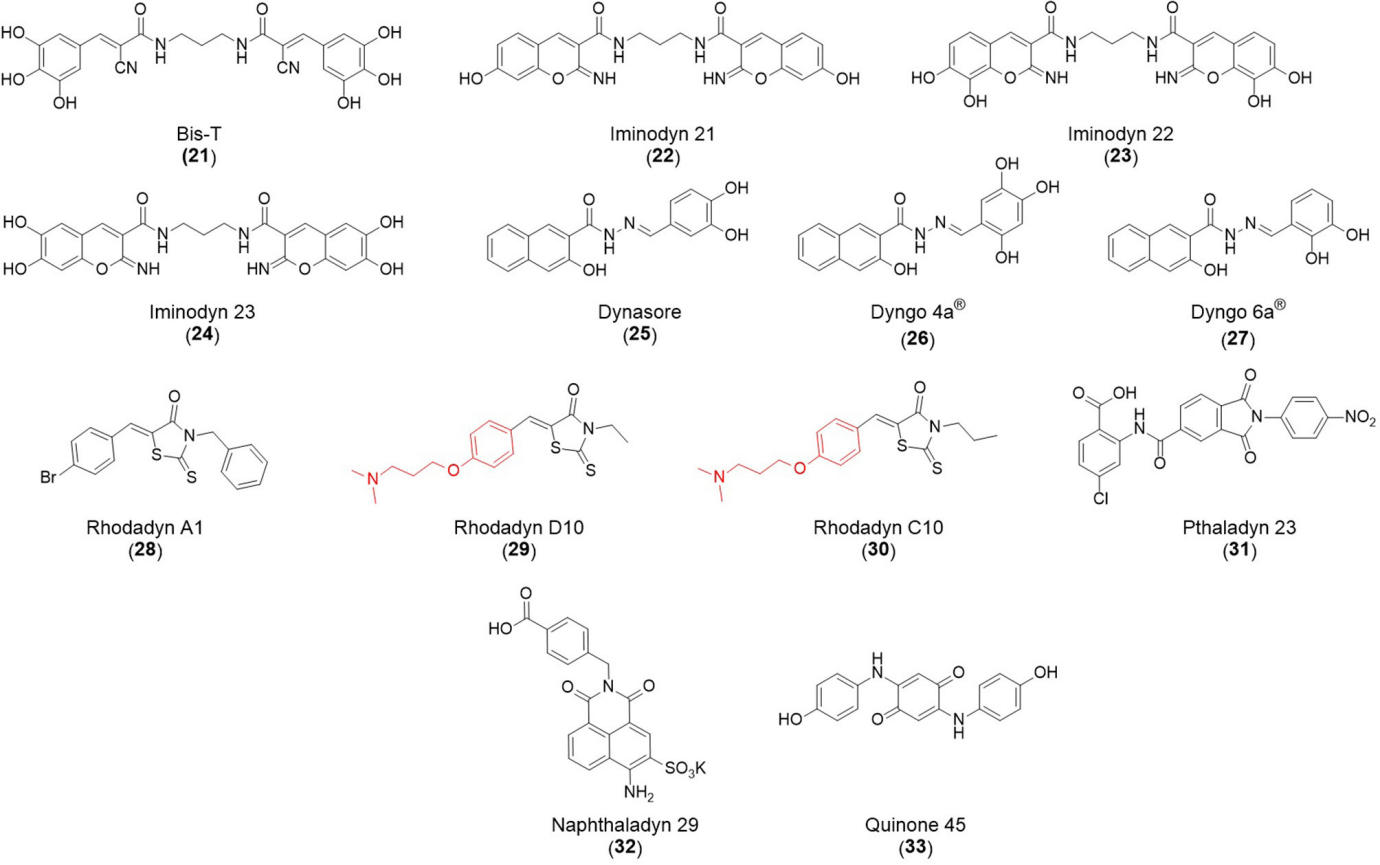
Structure of Bis-T (**21**) Iminodyn 21 **(22)**, Iminodyn 22 **(23)**, Iminodyn 23 (**24**), Dynasore (**25**), Dyngo 4a® (**26**), Dyngo 6a® (**27**), Rhodadyn A1 (**28**), Rhodadyn D10 (**29**), Rhodadyn C10 (**30**), Pthaladyn 23 (**31**), Naphthaladyn 29 (**32**), and Quinone 45 (**33**). The common N,N-dimethyl-3-phenoxypropan-1-amine head group of Rhodadyn D10 (**29**) and Rhodadyn C10 (**30**) is shown in red.

Bis-T (**21**) (Figure 11) is the most potent of the dimeric tryphostins (tyrosine phosphorylation inhibitors) reported as dynamin inhibitors, with an IC_50_ of 1.7 ± 0.2 μM (Hill et al., 2005). Structural modification to replace the nitrile group with a more drug like isostere, gave the Iminodyns, the first sub-micromolar potent dynamin inhibitors (Hill et al., 2010). The five most potent in this series were investigated further. Imidodyn 22 (**23**) inhibited the endocytosis (CME) of Texas Red-Transferrin (Tf-TxR) in human bone osteosarcoma epithelial cells (U2OS), with an IC_50_ of 10.7 ± 4.5 μM (Hill et al., 2010). Significant SVR inhibition of styryl dye FM4-64 uptake, with Iminodyn 21 (**22**), 22 (**23**) and 23 (**24**) (Figure 11) was noted, with Iminodyn 23 (**24**) the most potent (IC_50_ 40.4 ± 08 μM). Michaelis-Menten kinetics suggested that Iminodyn 22 (**23**) is an uncompetitive inhibitor of dynamin 1 (Hill et al., 2010). Iminodyn 22 (**23**) has since been used as a tool to investigate the role of dynamin in a range of processes, including elucidating new dynamin interactions [e.g., Nitric oxide synthase 1 (NOS1β)], dynamins role in phagosome scission and F-actin (filamentous) assembly and in the investigation viral cellular entry (dengue virus in macrophages) (Ayala-Nunez et al., 2016; Hyndman et al., 2016; Marie-Anaïs et al., 2016; Muranen et al., 2017).

### Dynasore and Dyngo 4a®

Macia *et al* identified Dynasore (**25**) (Figure 11), as a fast-acting, cell permeable, non-competitive small molecule inhibitor of dynamin 1 and 2 (Macia et al., 2006). The mechanism of action is unknown, Dynasore (**25**) did not affect GTP binding, dynamin self-assembly or oligomerisation but did disrupt dynamin dependent endocytic functions of the cell. Dynasore (**25**) arrested the formation of clathrin coated pits and vesicles and has since been used as a chemical probe to analyse the effect of dynamin on endocytosis in cells and inhibitory effects of dynamin *in vitro* (Kirchhausen et al., 2008). Unfortunately, Dynasore (**25**) was limited by non-specific binding properties. Binding to serum proteins resulted in a loss of inhibition. Further Dynasore (**25**) binds to trace levels of detergents, such as Tween-80, which are commonly used in biological assays (Mccluskey et al., 2013).

McCluskey et al. developed a second generation library of Dynasore (**25**) analogues with the objective to reduce non-specific binding and cytotoxicity. Dyngo 4a® (**26**) and Dyngo 6a® (**27**) (Figure 11), dynamin 1 IC_50_s of 0.38 ± 0.05 μM and 3.2 ± 0.3 μM, respectively [~30 times more potent than Dynasore (**25**) which has a dynamin 1 IC_50_ of 12.4 ± 1.5 μM], with reduced non-specific binding and cytotoxicity relative to Dynasore (Mccluskey et al., 2013). Unlike Dynasore (**25**), these compounds did not display off target protein-protein interactions with amphiphysin/clathrin or AP-2 and displayed a preference for the helical assembled dynamin, although there is still some debate regarding the specificity of the dyngo compounds (Park et al., 2013; Basagiannis et al., 2017; Persaud et al., 2018). This can, in part be off-set through the use of the inactive control compound, Dyngo-ϕ. Dyngo 4a® (**26**) inhibits SVE and activity-dependent bulk endocytosis (ADBE) at presynaptic nerve terminal (Mccluskey et al., 2013).

### Rhodadyns

Selective optimization of the side activities of a lead from the Pitstop® 2 (**10**) family afforded the rhodadyns as dynamin inhibitors (no clathrin inhibition off-target activity). Focused library development based on Rhodadyn A1 (**28**) (dynamin 1 IC_50_ 134 ± 21 μM), resulted in 13 analogues with dynamin 1 IC_50_s of ≤ 10 μM (dynamin 1) (Robertson et al., 2012). CME inhibition was also analysed using Texas red uptake assay. The most potent, Rhodadyn D10 (**29**), blocked Texas red uptake in U2OS cells with an IC_50_ of 5.9 ± 1.0 μM (dynamin 1 IC_50_ 4.5 ± 0.8 μM). Common to the two most potent analogues [Rhodadyn C10 (**30**) and D10 (**29**) (Figure 11)] and to the dynole and pyrimidine series of compounds was the *N,N*-dimethyl-3-aminopropan-1-amine head group both contained (shown in red in Figure 11) (Rhodadyn C10 (**30**) dynamin 1 IC_50_ 7.1 ± 1.0 μM). The introduction of a terminal carboxylate moiety abolished CME activity (Robertson et al., 2012).

### Naphthaladyns and Pthaladyns

The Naphthaladyns and Pthaladyns were the first classes of dynamin inhibitor targeting the GTPase domain to be rationally designed. The Pthaladyns were developed from virtual screening lead of a homology model of the human dynamin 1 GTPase domain (Odell et al., 2010; Macgregor et al., 2014a; Abdel-Hamid et al., 2015). No pthaladyn-based dynamin 1 inhibitor blocked CME. This was attributed to poor cell permeability or their rapid degradation (Odell et al., 2010). Pthaladyn 23 (**31**) did return an SVE IC_50_ of 12.9 ± 5.9 μM (FM4-64 uptake in synaptosomes), similar to the dynamin 1 inhibition observed for this compound (IC_50_ = 17.4 ± 5.8 μM) (Odell et al., 2010). However, in an assay examining the effects of pthaladyn 23 (**31**) in synaptosomes, an IC_50(SVE)_ of 743 μM was observed (Daniel et al., 2012).

Modelling led side activity optimization of the 1,8-naphthalimide scaffold found in the Pitstop® 1 (**9**) family, via imide modification resulted in the development of a library of Naphthaladyns (Macgregor et al., 2014a; Abdel-Hamid et al., 2015). Naphthaladyn 29 (**32**) (Figure 11), displayed a dynamin 1 IC_50_ of 18.5 ± 1.7 μM, a CME IC_50_ of 66 μm (inhibition of Tf-a594 internalisation in U2OS cells) inhibition of internalisation and was GTP domain competitive (Abdel-Hamid et al., 2015).

### Quinodyns

Also identified by *in silico* screening, as with the Pthaladyns, the Quinodyns arose from the same *in silico* library screening of the human dynamin GTPase domain model that afforded the Pthaladyns. Dynamin activity was confirmed using a minimal dynamin construct (containing the GTPase domain and BSE) (Chappie et al., 2011; Macgregor et al., 2014b). Quinone 45 (**33**) (Figure 11) inhibited GTPase dynamin 1 (IC_50_ of 11.1 ± 3.6 μm) and inhibited transferrin uptake with an IC_50_ of 36 ± 16 μm (Macgregor et al., 2014b).

### Pyrimidyns

**Figure 12:**
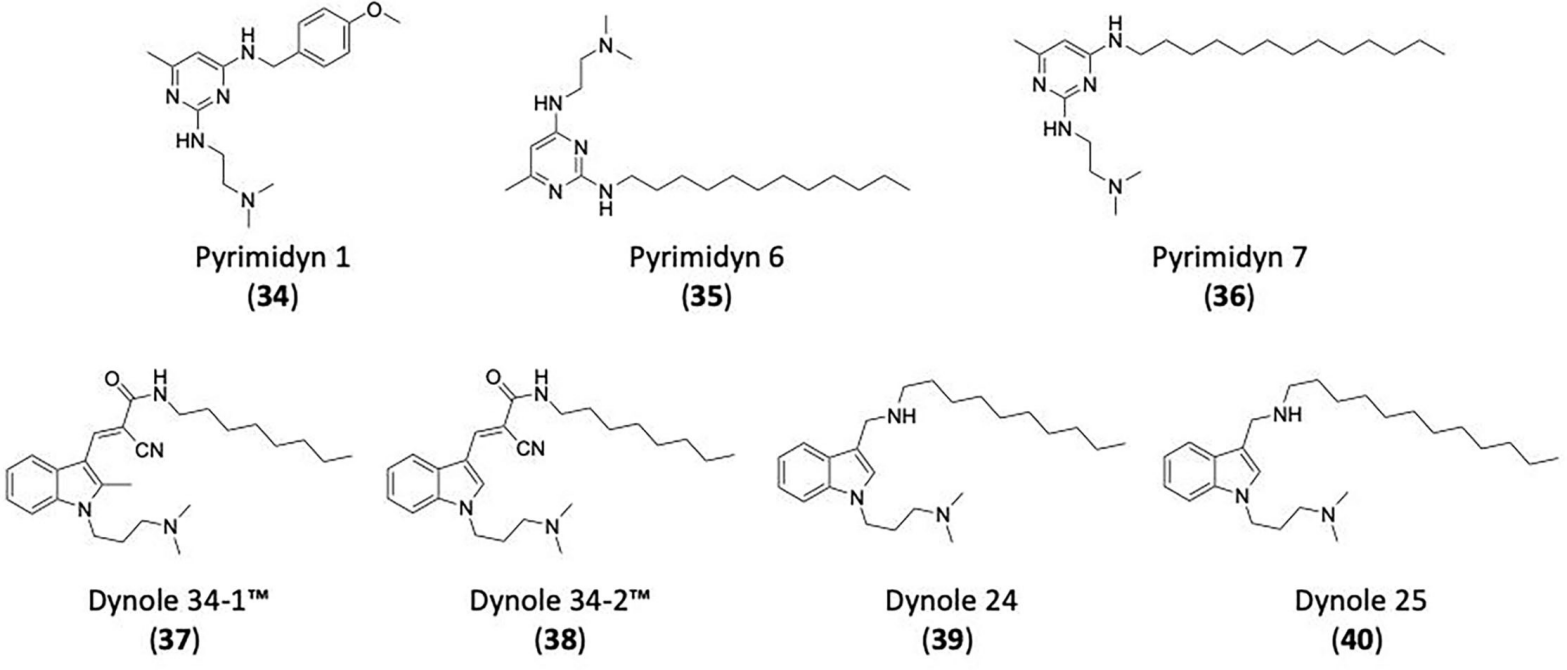
Structures of Pyrimidyn 1 (**34**), Pyrimidyn 6 (**35**), Pyrimidyn 7 (**36**), Dynole 34-1™ (**37**), Dynole 34-2™ (**38**), dynole 24 (**39**), and dynole 25 (**40**) are shown.

A small library of substituted pyrimidines, the Pyrimidyns, were designed based on library screening and resulted in the identification of Pyrimidyn 1 (**34**) as a modest dynamin 1 inhibitor (IC_50_ 35.3 ± 7.1 μM) (McGeachie et al., 2013). The two most potent compounds from subsequent focused library development showed a 30-fold increase in dynamin 1 inhibition, with Pyrimidyn 6 (**35**) and Pyrimidyn 7 (**36**) (IC_50_s of 1.4 ± 0.2 μM and 1.1 ± 0.05 μM, respectively) (Figure 12) (McGeachie et al., 2013). Both Pyrimidyn 6 (**35**) and Pyrimidyn 7 (**36**) were found to obstruct interaction of dynamin with phospholipids, and also the binding of GTP to dynamin concluding that they target two different domains simultaneously, the GTPase and PH domain, via a dual mechanism of action. Pyrimidyn 6 (**35**) and Pyrimidyn 7 (**36**) were also found to have a potent inhibitory action against CME with IC_50_ values of 19.3 ± 3.5 μM and 12.1 ± 2.1 μM, respectively (McGeachie et al., 2013).

### Dynoles®

The Dynoles™ are a class of dynamin inhibitors, designed by structural simplification of the lead, staurosporine. This resulted in structures with a single indole and an alkyl dimethyl amino moiety. This initial library produced only moderate dynamin 1 inhibitors, with the introduction of a C-3 alkyl chain yielding a 10–30-fold potency increase (Hill et al., 2009). The most active compound in the series, Dynole 34-1™ (**37**) (dynamin 1 IC_50_ = 3.33 ± 0.75 μM) was highly lipophilic (Figure 12). It was hypothesised that removal of the C2-methyl of Dynole 34-1™ (**37**) would result a more favourable LogP, while retaining activity. This gave Dynole 34-2™ (**38**) (Figure 12), with a 3-fold increase in potency (IC_50_ of 1.30 ± 0.30 μM) (Hill et al., 2009). A second generation of Dynole compounds, elicited two compounds, dynole 24 (**39**) and dynole 25 (**40**) (Figure 12) that displayed greatly reduced cell toxicity, with sub-micromolar potency against dynamin 1 (IC_50_ 0.56 ± 0.09 μM and 0.76 ± 0.05 μM, respectively) (Gordon et al., 2013). In-cell testing for CME (mediated by dynamin 2) showed compound dynole 24 (**39**) had an IC_50_ 3 times more potent that Dynole 34-2™ (**38**) [IC_50_ of 1.9 ± 0.3 μM for dynole 24 (**39**) compared to 5.0 ± 0.9 μM for Dynole 34-2™ (**38**)] (Gordon et al., 2013). Dynole 24 (**39**) was also found to be 4.4-fold more selective towards dynamin 1 over dynamin 2 (compared to Dynole 34-2™ (**38**) which displays 2-fold selectivity for dynamin 1) (Gordon et al., 2013).

### Bisphosphonates

**Figure 13:**
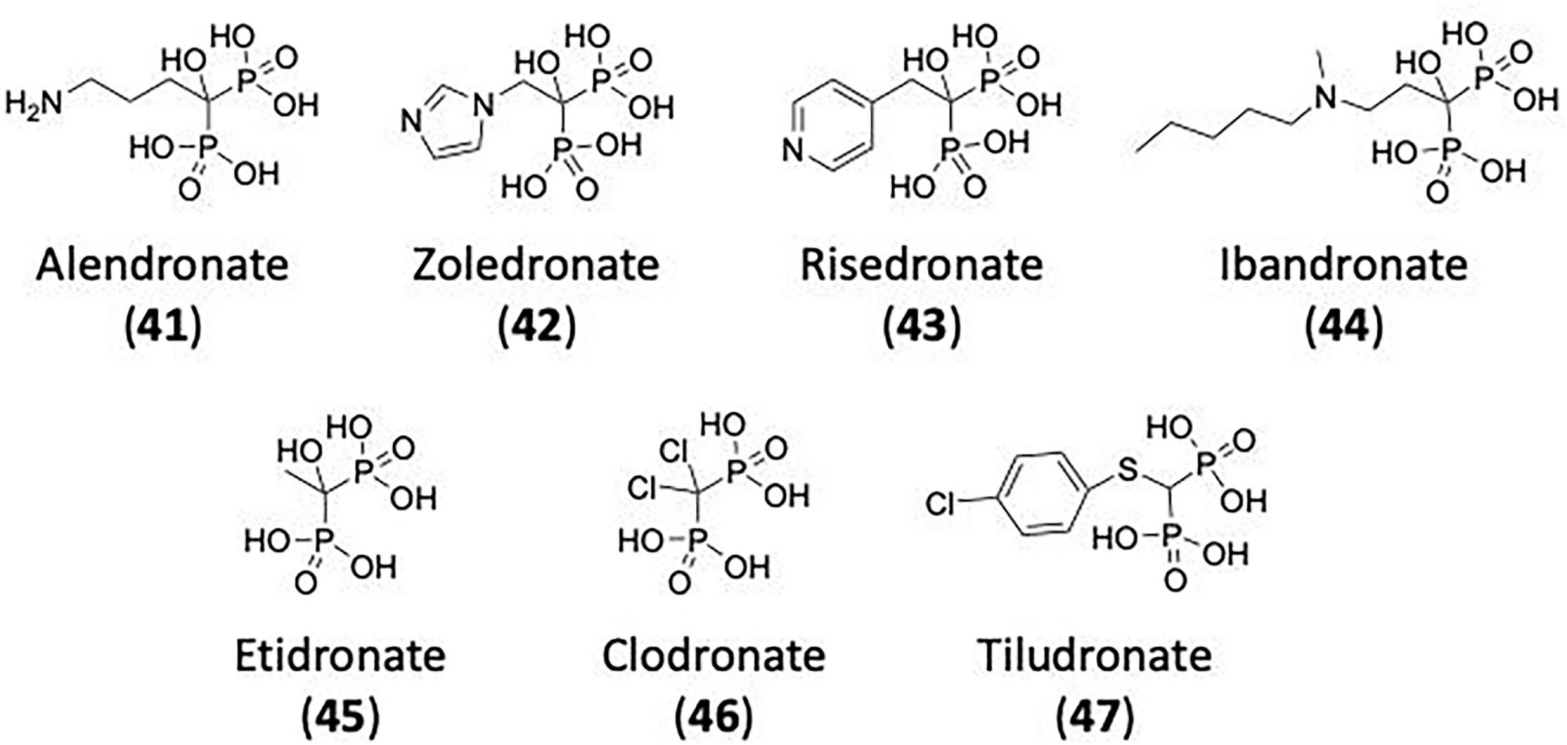
Bisphosphonates (BPs) as dynamin inhibitors. Nitrogen containing BPs (N-BPs); alendronate (**41**), zoledronate (**42**), risedronate (**43**), and ibandronate (**44**) and Non-nitrogen containing BPs (NN-BPs); etidronate (**45**), clodronate (**46**) and tiludronate (**47**). Alendronate (**41**), zoledronate (**42**), and etidronate (**45**) are believed to inhibited CME via dynamin inhibition.

Bisphosphonates (BPs) are synthetic analogues of phyrophosphate, which can be sub-classified into two classes, depending on the presence or absence of a nitrogen in the structure; non-nitrogen containing BPs (NN-BPs) and nitrogen containing BPs (N-BPs) (Reszka and Rodan, 2003). Some compounds in this class are used as prescriptions drugs against the loss of bone mass arising from a range of medical conditions (Maraka and Kennel, 2015). N-BPs, such as alendronate (**41**), zoledronate (**42**), risedronate (**43**), and ibandronate (**44**) are the most widely prescribed, however some NN-BPs are also used, including etidronate (**45**), clodronate (**46**) and tiludronate (**47**) (Figure 13) (Maraka and Kennel, 2015). Dynamin 2 was identified as a possible target for BP drugs, and it was determined that three different BPs, alendronate (**41**), zoledronate (**42**), and etidronate (**45**), inhibited the uptake of transferrin as well as adenovirus and simian virus 40 (Masaike et al., 2010). Titration experiments with acidic phospholipids indicated BPs bind to the PH domain, however the proposed mechanism is still debated (Masaike et al., 2010; Eschenburg and Reubold, 2018).

## Rationalising Clathrin and Dynamin Inhibitor Potency

In terms of clathrin inhibitory potency, the co-crystal structures of Pitstop® 1 and Pitstop® 2 and the clathrin terminal domain enables the identification of the key residues involved in inhibitor binding. The inhibition of the CTD interaction with amphiphysin 2 depends on the disruption of a protein-protein interaction. This more complex than traditional small molecule inhibitor development as the protein-protein interface is typically large and shallow. This is further complicated within site 1—the Pitstop® binding site which is lined predominately with hydrophobic residues: I52, I62, I80, I93, L82 and F91 (Figures 8A,C,D). Most probably, increased binding would most result from increased analogue hydrophobicity, which is counter to the introduction of drug like properties. Alternatively, given that Pitstop® 1 and Pitstop® 2 bind in the same site, but offset to each other, chimeric analogues are possible, but these will still, most likely, suffer from increased hydrophobicity, and in this instance, increased molecular weights again pushing these compounds out with the physicochemical properties associated with drugs.

In terms of drug development, dynamin inhibitors appear to offer greater scope for development. There have been multiple inhibitors across multiple chemical scaffolds and modes of dynamin inhibition reported. Of those reported to date, those targeting the PH domain are probably the least likely to be developed to clinical candidates. Despite CEREP ExpresS screening showing limited problematic off target effects, the wide spread prevalence of PH domains in proteins raises the spectre of off-target effects that would be difficult to design away from and gain dynamin selectivity. The efficacy of APDs against dynamin is typically at a level 100x higher (as is the efficacy of SSRI's) than the primary protein targets, and while a number of these analogues are known to accumulate and can be found at high concentrations, the difference between primary and off-target effect (dynamin) renders their therapeutic use for endocytosis modulation highly questionable. However, it is always important to know that the side-effect severity is associated with the extent of the medical need.

The *in vivo* efficacy noted with dynole 34-2 suggests that on-going development in this family of compounds may generate a true drug lead, but while tolerated and synergistic with current chemotherapy regimes in mouse models of T-ALL and MAL, the current lack of known binding site within dynamin limits the potential for a rational drug design approach. While efficacious in a cytotoxicity trial, this does not automatically translate to prophylactic or chronic exposure safety and efficacy. The Bis-T and Dyngo series of compounds are also limited the same lack of knowledge with respect to dynamin binding site.

Most promising are those analogues that target the dynamin GTPase domain. As noted this domain is significantly larger than that observed with traditional Ras-like GTPases, as such protein selectivity is theoretically achievable. To date Dynamin GTPase competitive analogues have been reported across three chemical scaffolds: the pthaladyns, Naphthaladyn and Quinodyns. The latter, Quinodyns, contain a promiscuous quinone moiety and while analogues of this nature are clinically used, they are difficult compounds too develop due to their potential off-target effects. The SAR around both the Pthaladyns and Naphthaladyns demonstrate that increased efficacy is feasible. Recent structural reports of the dynamin crystal structure and more importantly the cryo-EM structure of fully assembled helical dynamin permit more accurate rational design and virtual screening programs to be developed. The modelling approach, with the Pthaladyns and Naphthaladyns led the introduction of key structural motifs that saw increases in potency across both scaffolds of 10–20-fold. Activity increases were evident in the introduction of substituted amides and substituted N-imides with the Pthaladyns. The removal of naphthalene substituents and the introduction of aromatic imides with the Naphthaladyns removed clathrin inhibition while enhancing dynamin potency.

## Conclusions

In this review, the process of CME has been discussed in order to illuminate the function of two key proteins; clathrin and dynamin. These proteins play essential roles with clathrin forming the being the main component of CCV and CCPs and dynamin required during scission, allowing the formed vesicle to pinch off from the membrane and travel to its destination. Although CME does not account for all SVR, this process does play a key role in the endocytic cycle upon which various neurological conditions rely (e.g., Parkinson's, Alzheimer's, epilepsy, and schizophrenia).

With no treatments currently available to cure neurodegenerative diseases investigating these endocytic processes, focusing on clathrin and dynamin provides new therapeutic targets for neurological diseases. General cellular perturbants along with genetic approaches to clathrin and dynamin inhibitors have been used to provide insight into the mechanisms of pathways, however for therapeutic use novel small molecule inhibitors seem the most auspicious approach. Building on the compounds discussed in this review, the development of new and potent clathrin and dynamin inhibitor is an extremely promising avenue for the development of new therapeutics targeting endocytosis, for treatments of neurological diseases.

A common comment relates to the potency of such analogues, here from sub-micromolar to micromolar and how this potency is not appropriate for their use in a clinical setting. Another is that endocytosis is a critical process, and inhibition by this criteria should be lethal. However, total inhibition of protein function is not the goal of a small molecule, more often these “drugs” seek to modulate protein activity enabling endogenous systems to regain balance, of systems are temporally inhibited only until the affected protein can be regenerated by the host system, e.g., in the case of covalent inhibitors. If this was not the case, protein kinase inhibitors and immunosuppressive agents would not be of clinical value. Moreover, it has been shown that transient endocytosis inhibition by Stemetil is tolerated and elicited a favourable response in ADCC adjuvant treatment with cetuximab in head and neck cancer.

These inhibitors may ultimately lead to the clinical use of endocytosis modulators in humans. While endocytosis is a critical mechanism in maintaining cell and thus host well-being, there is growing evidence that transient modulation is not only feasible but may have positive therapeutic outcomes. Chew et al. (2020) report on blocking CME as an effective strategy to improve the clinical response to antibody dependent cellular cytotoxicity mediating antibodies in humans; Tremblay et al. (2020) report that the dynamin inhibitor, dynole 34-4 is highly synergistic with existing chemotherapy in mouse models of T-ALL and AML; Powell et al. (2021) report that endocytosis inhibition attenuates inflammatory pain like behaviour; and Jensen et al. (2017) also reported on an endocytosis mediated effect as a viable therapeutic target for prolonged pain relief. While these findings should be considered positive, it is prudent to temper the enthusiasm that may be apparent with the realisation that the path to the clinic is long and winding. Multiple drug targets show such early-stage progress, but for a myriad of reasons fail to gain a foothold in a clinical setting. Time, money and extensive future studies will be required to deliver safe and efficacious CME inhibitors as therapeutics.

## Author Contributions

All authors listed have made a substantial, direct, and intellectual contribution to the work and approved it for publication.

## Funding

Funding from the Australian Research Council and the National Health and Medical Research Council (Australia) is gratefully acknowledged.

## Conflict of Interest

AM has a commercial compound supply agreement with Abcam (UK) which includes some of the compounds mentioned in this article. The remaining authors declare that the research was conducted in the absence of any commercial or financial relationships that could be construed as a potential conflict of interest.

## Publisher's Note

All claims expressed in this article are solely those of the authors and do not necessarily represent those of their affiliated organizations, or those of the publisher, the editors and the reviewers. Any product that may be evaluated in this article, or claim that may be made by its manufacturer, is not guaranteed or endorsed by the publisher.
